# Prevention of Cardiovascular Disease Events and Deaths Among Black Adults Via Systolic Blood Pressure Equity

**DOI:** 10.1001/jamanetworkopen.2025.41336

**Published:** 2025-11-04

**Authors:** Shakia T. Hardy, Lei Huang, Lisandro D. Colantonio, Oluwasegun P. Akinyelure, Kathryn Foti, Lama Ghazi, Timothy B. Plante, Monika M. Safford, Paul Muntner

**Affiliations:** 1 Department of Epidemiology, University of North Carolina at Chapel Hill, Chapel Hill; 2 Department of Epidemiology, University of Alabama at Birmingham, Birmingham; 3 Department of Medicine, Larner College of Medicine, University of Vermont, Burlington; 4 Department of Medicine, Weill Cornell Medicine, New York, New York; 5 Perisphere Real World Evidence, Austin, Texas

## Abstract

**Importance:**

There are disparities in blood pressure (BP) levels by race in the US.

**Objective:**

To estimate the number of cardiovascular disease (CVD) events and deaths that could be prevented among non-Hispanic Black adults by achieving the same mean systolic BP (SBP) as non-Hispanic White adults 45 years or older.

**Design, Setting, and Participants:**

In this modeling study, the difference in mean SBP between non-Hispanic Black and non-Hispanic White US adults was calculated from the 2015-2020 cycles of the National Health and Nutrition Examination Survey (NHANES). The 10-year cumulative incidence of CVD, including stroke, coronary heart disease, and heart failure, and CVD mortality for non-Hispanic Black adults were calculated using data from the Reasons for Geographic and Racial Differences in Stroke (REGARDS) study. The relative risk reduction for CVD events and mortality with initiation and intensification of antihypertensive medication treatment was applied from the Blood Pressure Lowering Treatment Trialists Collaboration (BPLTTC), calibrated to reflect the SBP reduction for non-Hispanic Black adults to have the same SBP as non-Hispanic White adults. Data were analyzed from June 22, 2022, to August 13, 2025.

**Exposure:**

Systolic blood pressure.

**Main Outcomes and Measures:**

Number of CVD events and deaths. Using data from NHANES, REGARDS, and BPLTCC, the cumulative incidence of CVD events and deaths expected over the next 10 years with current SBP levels and with SBP equity was estimated.

**Results:**

Among 82.3 million US adults included in the study, 37.2 million (45.3%) were men, 45.0 million (54.7%) were women, 10.1 million (12.2%) were non-Hispanic Black, and 72.2 million (87.8%) were non-Hispanic White. The mean (SD) age was 60.8 (0.3) years. The mean SBP was 130.7 (95% CI, 129.0-132.5) mm Hg among non-Hispanic Black adults and 124.2 (95% CI, 123.1-125.3) mm Hg among non-Hispanic White adults for those not taking antihypertensive medication (difference, 6.5 [95% CI, 4.5-8.5] mm Hg), and 137.8 (95% CI, 135.8-139.8) mm Hg among non-Hispanic Black adults and 131.2 (95% CI, 129.7-132.7) mm Hg among non-Hispanic White adults for those taking antihypertensive medication (difference, 6.5 [95% CI, 4.0-9.1] mm Hg). Achieving equity in SBP between non-Hispanic Black and White adults was projected to reduce the number of CVD events during 10 years by 50 434 (95% CI, 33 985-71 137) among non-Hispanic Black US adults not taking antihypertensive medication and 122 881 (95% CI, 83 220-176 826) among non-Hispanic Black adults taking antihypertensive medication. Achieving equity in SBP between non-Hispanic Black and White adults was projected to reduce the number of CVD deaths during 10 years by 21 703 (95% CI, 7313-40 278) among non-Hispanic Black US adults not taking antihypertensive medication and 55 055 (95% CI, 19 823-99 693) among non-Hispanic Black adults taking antihypertensive medication. The largest proportion of CVD events and deaths prevented were among those aged 45 to 64 years. For example, more than half of CVD events prevented among non-Hispanic Black adults not taking antihypertensive medication (55%) were in that age group.

**Conclusions and Relevance:**

The findings of this modeling study suggest that achieving SBP equity between non-Hispanic Black and White adults could substantially reduce the number of CVD events and deaths experienced by non-Hispanic Black US adults. Initiatives to maintain normal BP and achieve BP control for individuals with hypertension could have a substantial impact on health equity in the US.

## Key Points

**Question:**

What number of cardiovascular disease (CVD) events and deaths could be prevented among US non-Hispanic Black adults if their mean systolic blood pressure (SBP) were lowered to equal that of non-Hispanic White adults?

**Findings:**

In this modeling study of 10.1 million US Black adults, achieving SBP equity was projected to prevent more than 170 000 CVD events and 75 000 CVD deaths during 10 years among non-Hispanic Black adults 45 years or older, with more than half of the reductions occurring in individuals aged 45 to 64 years.

**Meaning:**

These findings suggest that achieving SBP equity between Black and White adults could substantially reduce CVD burden and may have a substantial impact on equity-focused prevention.

## Teaser

This modeling study estimates the number of cardiovascular disease (CVD) events and deaths that could be prevented among non-Hispanic Black adults in the US by achieving systolic blood pressure (SBP) equal to that of non-Hispanic White adults.

## Introduction

High blood pressure (BP) contributes to more cardiovascular disease (CVD) events and deaths than any other modifiable risk factor in the US and worldwide.^1,2,3^ Randomized clinical trials have demonstrated the efficacy of nonpharmacologic interventions and antihypertensive medication for lowering BP and preventing CVD.^4,5^ Despite the availability of approaches to reduce BP among all racial and ethnic groups, racial disparities in systolic BP (SBP) have persisted for decades, contributing to a higher rate of CVD events and mortality among non-Hispanic Black compared with non-Hispanic White US adults.^6,7,8^

Disparities in hypertension and BP control can be eliminated. A prior analysis of the Reasons for Geographic and Racial Differences in Stroke (REGARDS) study^9^ found that modifiable social and behavioral factors explained most of the higher incidence of hypertension among Black compared with White adults. In the Systolic Blood Pressure Intervention Trial (SPRINT),^10^ which included adults with hypertension, non-Hispanic Black and White participants achieved comparable SBP levels and the same reduction in CVD after intensive antihypertensive medication treatment to a goal of 120 mm Hg or less.

The purpose of the present study was to estimate the number of CVD events and deaths that could be prevented among non-Hispanic Black adults by achieving SBP equity (ie, the same mean SBP as experienced by non-Hispanic White adults in the US). To accomplish this goal, we analyzed data from the National Health and Nutrition Examination Survey (NHANES) as the reference standard for nationally representative BP data, the REGARDS study for contemporary estimates of CVD incidences in the US, and published estimates of the reduction in CVD events and CVD mortality with BP lowering from the Blood Pressure Lowering Treatment Trialists Collaboration (BPLTTC).^11^

## Methods

### Data Sources

This modeling study followed the Strengthening the Reporting of Observational Studies in Epidemiology (STROBE) reporting guideline. NHANES was designed to assess the health and nutritional status of the noninstitutionalized US population. Since the 1999-2000 cycle, the National Center for Health Statistics has conducted NHANES in 2-year cycles using a multistage probability sampling design.^12^ The publicly available data files for the NHANES 2015-2016 and 2017-2020 cycles were pooled for the current analysis. The National Center for Health Statistics Institutional Review Board approved the study protocol for each NHANES cycle, and written informed consent was obtained from each participant.

The REGARDS study enrolled a population-based cohort of 30 239 non-Hispanic Black and White US adults 45 years or older from the 48 contiguous US states and Washington, DC.^13^ The REGARDS study participants completed a baseline visit between 2003 and 2007 and have been followed up for CVD events and mortality. The REGARDS study was approved by the institutional review boards of all participating institutions, and all participants provided written informed consent.

The BPLTTC has published estimates of the reduction in CVD events and mortality rates associated with 5–mm Hg lower SBP through antihypertensive medication treatment using individual participant data from more than 50 large-scale outcome trials.^11^ Estimates from participants without a history of CVD were used in the present analysis. The BPLTTC obtained ethics review approval from the Oxford Central Ethics University Research Committee.

### Data Collection

#### NHANES

NHANES data were collected during an in-home interview and a study visit at a mobile examination center. Of relevance to the current analysis, age, sex, race and ethnicity, income-to-poverty ratio, type of health insurance, smoking status, alcohol intake, and physical activity were self-reported and assessed using standardized questionnaires. Using calibrated equipment, height and weight were measured during the NHANES examination following a standardized protocol. Body mass index was calculated as weight in kilograms divided by height in meters squared. Glycated hemoglobin level was measured using standard methods. Diabetes was defined as glycated hemoglobin levels of 6.5% or greater or self-reported use of insulin or oral medication to lower glucose levels.

Trained physicians measured BP following a standardized protocol. After 5 minutes of seated rest, as many as 3 BP measurements were obtained at 30-second intervals using an appropriately sized BP cuff and a mercury sphygmomanometer in the NHANES 2015-2016 cycle and a validated oscillometric device in the NHANES 2017-2020 cycle. Based on the evaluation of the differences in mean SBP and diastolic BP (DBP) when measured by the 2 devices, we calibrated the oscillometric device values to the mercury device values by adding 1.5 mm Hg to oscillometric-measured SBP and subtracting 1.3 mm Hg from oscillometric-measured DBP for participants in the NHANES 2017-2020 cycle.^14^

NHANES study participants who were 45 years or older, attended the interview and examination, had at least 1 SBP and 1 DBP measurement, had self-reported information on antihypertensive medication use, and had self-reported being non-Hispanic Black or non-Hispanic White were included in the analysis (eFigure 1 in Supplement 1). We excluded participants who self-reported a history of CHD, stroke, or heart failure.

#### REGARDS Study

At baseline, data were collected by trained research staff through computer-assisted telephone interviews and by trained technicians during a subsequent in-home study visit.^13^ REGARDS study participants were included in this analysis if they had at least 1 SBP and 1 DBP measurement at baseline, had self-reported information on antihypertensive medication use and follow-up data for outcomes, and had no CVD, including self-reported CHD, stroke, or suspected heart failure at baseline. The final sample size was 28 574 participants (eFigure 2 in Supplement 1).^15^

During twice-annual phone calls, participants or their proxies were asked to report hospitalizations, and proxies were asked to report details surrounding the deaths of participants. Medical records for suspected heart disease or stroke hospitalizations were retrieved for adjudication. Expert clinician adjudication of CVD events in the REGARDS study, including fatal or nonfatal stroke, CHD defined by fatal and nonfatal myocardial infarction or CHD death, and fatal or nonfatal heart failure hospitalizations, have been described previously and followed national guidelines.^16^

#### BPLTTC

We abstracted the relative risk (RR) for CVD, including stroke, CHD, or heart failure, and CVD mortality associated with a 5–mm Hg reduction in SBP with initiation and intensification of antihypertensive medication use among those without a history of CVD from the BPLTTC publication.^11^ The BPLTTC defined CHD as fatal or nonfatal myocardial infarction or ischemic heart disease, stroke as a composite of fatal or nonfatal stroke, and heart failure as death or hospital admission due to heart failure.

### Statistical Analysis

Data were analyzed from June 22, 2022, to August 13, 2025. All analyses were stratified by age (45-64, 65-74, and ≥75 years), race and ethnicity, and sex. Using NHANES 2015-2020 data, we estimated the number, characteristics, and, separately, mean SBP and DBP for non-Hispanic Black and White US adults who were and were not taking antihypertensive medication.

Using data from the REGARDS study, we estimated the overall and age- and sex-specific 10-year cumulative incidence of CVD, stroke, CHD, heart failure, and CVD mortality among non-Hispanic Black adults who were and were not taking antihypertensive medication. For analyses of CVD, stroke, CHD, and heart failure, we accounted for the competing risk of death using a semiparametric Fine-Gray model.^17^ For analyses of CVD mortality, we accounted for the competing risk of non-CVD mortality.

The following analyses were completed separately for those not taking and taking antihypertensive medication. The analysis for US adults not taking antihypertensive medication is detailed later in this section and was repeated for US adults taking antihypertensive medication. We estimated the number of CVD events expected to occur during 10 years among non-Hispanic Black US adults with their current SBP levels. To do so, we calculated the number of US non-Hispanic Black adults not taking antihypertensive medication within each age-sex subgroup from the NHANES 2015-2020 cycles. We multiplied the number of US Black adults not taking antihypertensive medication within age and sex subgroup by the age- and sex-specific 10-year cumulative incidence of CVD from the REGARDS study. We summed the number of events across age and sex subgroups to estimate the total number of CVD events expected with current SBP levels among non-Hispanic Black adults.

We conducted the following 2 steps to estimate the number of CVD events expected during the next 10 years among US Black adults if SBP equity was achieved. First, we calibrated the RR for CVD from the BPLTTC to reflect the difference in SBP between Black and White adults from NHANES 2015-2020 cycles using the following equation:

RR Calibrated = exp(ln[RR for CVD with 5–mm Hg reduction in SBP from the BPLTTC] × [mean SBP among non-Hispanic Black US adults − mean SBP among non-Hispanic White US adults from NHANES]/5).

Second, within each age and sex subgroup, we multiplied the number of non-Hispanic Black US adults not taking antihypertensive medication by the 10-year cumulative incidence of CVD among non-Hispanic Black adults and the calibrated RR estimated in step 1.

Among non-Hispanic Black adults, we subtracted the number of CVD events expected with SBP equity from the number of CVD events expected with current SBP levels (ie, the natural course). We summed the number of CVD events across age and sex subgroups to estimate the total number of CVD events expected with SBP equity among non-Hispanic Black adults. We estimated 95% CIs for the projected number of preventable cardiovascular events using 1000-iteration Monte-Carlo simulations. In each iteration, we sampled from the assumed normal distributions of the NHANES estimates, 10-year cumulative incidence estimates, and RRs from the BPLTTC and reran the aforementioned analytic steps. Bias-corrected 95% CIs were obtained from the resulting distributions. These analyses were repeated for the following outcomes: stroke, CHD, heart failure, and CVD mortality. Given potential issues with transportability of the RRs from the BPLTTC to the US population, we conducted a sensitivity analysis that assumed a 10% increase and 10% decrease in the effectiveness of BP lowering on CVD events among the US population compared with the BPLTTC population. The analysis of NHANES data accounted for the multistage approach to selecting participants and were weighted to provide estimates for the noninstitutionalized US population. All analyses were conducted using SAS, version 9.2 (SAS Institute Inc), and R, version 4.3.2 (R Program for Statistical Computing).

## Results

A total of 82.3 million US adults were included in the study; 37.2 million (45.3%) were men, 45.0 million (54.7%) were women, 10.1 million (12.2%) were non-Hispanic Black, and 72.2 million (87.8%) were non-Hispanic White. The mean (SD) age was 60.8 (0.3) years. In the NHANES 2015-2020 cycles, non-Hispanic Black adults not taking and taking antihypertensive medication were more likely than non-Hispanic White adults to be 45 to 64 years of age vs 65 to 74 years or 75 years and older; to have obesity, diabetes, and an income-to-poverty ratio less than 1.30; to be current smokers; and not to consume alcohol. Non-Hispanic Black adults were less likely than non-Hispanic White adults to have at least 150 minutes of physical activity per week (Table 1).

**Table 1.** Characteristics of Non-Hispanic Black and Non-Hispanic White US Adults by Use of Antihypertensive Medication in the National Health and Nutrition Examination Survey 2015-2020 Cycles

Characteristic	Not taking antihypertensive medication, % (95% CI)		Taking antihypertensive medication, % (95% CI)	
	Non-Hispanic White adults (n = 1330)	Non-Hispanic Black adults (n = 688)	Non-Hispanic White adults (n = 887)	Non-Hispanic Black adults (n = 824)
Age, y				
45-64	71.5 (67.3-75.4)	81.2 (77.9-84.2)	48.3 (44.4-52.1)	64.1 (60.1-68.0)
65-74	19.0 (15.7-22.7)	12.5 (9.6-15.8)	31.4 (27.0-36.0)	22.1 (19.3-25.1)
≥75	9.5 (7.8-11.4)	6.3 (4.6-8.4)	20.3 (17.7-23.1)	13.7 (11.0-16.8)
Sex				
Men	46.3 (43.6-49.0)	48.5 (44.5- 52.5)	44.1 (40.1- 48.1)	38.7 (35.2-42.3)
Women	53.7 (51.0-56.4)	51.5 (47.5-55.5)	55.9 (51.9-59.9)	61.3 (57.7-64.8)
BMI				
Healthy weight (<25)	29.9 (26.2-33.8)	25.5 (21.7-29.6)	14.5 (11.8-17.5)	14.0 (11.2-17.1)
Overweight (25-29.9)	37.7 (34.3-41.3)	34.2 (30.4-38.0)	32.1 (27.4-37.0)	25.0 (22.0-28.2)
Obesity (≥30)	32.4 (29.7-35.1)	40.3 (36.4-44.4)	53.4 (49.0-57.8)	61.0 (57.0-65.0)
Diabetes	8.0 (6.0-10.5)	12.0 (9.7-14.7)	24.0 (21.2-27.0)	35.3 (31.8-38.9)
Poverty-to-income ratio				
<1.30	8.9 (7.1-10.9)	26.1 (21.4-31.2)	11.0 (8.4-14.1)	31.3 (27.6-35.1)
1.30-3.49	26.8 (22.9-31.0)	40.6 (35.7-45.8)	36.2 (32.5-39.9)	36.0 (31.6-40.6)
≥3.50	64.4 (60.1-68.5)	33.3 (27.3-39.7)	52.8 (48.1-57.5)	32.7 (26.8-39.2)
Health insurance				
Private	55.0 (50.3-59.6)	43.8 (37.7-49.9)	36.5 (32.4-40.8)	32.9 (28.2-38.0)
Medicare	21.8 (18.6-25.3)	16.1 (13.4-19.1)	38.1 (34.2-42.1)	23.6 (20.1-27.4)
Medicaid	3.6 (2.5-4.9)	12.5 (8.9-16.9)	4.7 (3.2-6.6)	17.4 (14.0-21.1)
Other government	13.4 (10.7-16.3)	13.0 (9.7-16.9)	17.3 (14.3-20.7)	18.3 (15.6-21.2)
Uninsured	6.2 (4.4-8.6)	14.6 (12.0-17.5)	3.4 (2.2-4.8)	7.8 (5.8-10.1)
Current smoking	13.2 (11.4-15.3)	23.7 (20.0-27.7)	13.2 (10.0-16.9)	20.1 (17.1-23.5)
Alcohol consumption				
Heavy	9.2 (7.0-11.9)	7.5 (5.1-10.7)	7.8 (5.6-10.5)	6.8 (5.2-8.9)
Moderate	67.2 (63.2-70.9)	62.1 (58.1-66.0)	62.9 (57.4-68.0)	53.2 (49.1-57.3)
None	23.6 (20.5-26.9)	30.4 (26.6-34.4)	29.4 (24.9-34.2)	40.0 (36.3-43.7)
≥150 min of Physical activity per week	67.2 (64.2-70.0)	52.7 (48.9-56.5)	53.4 (48.5-58.2)	48.3 (43.8-52.9)
SBP, mean (95% CI), mm Hg	124.2 (123.1-125.3)	130.7 (129.0-132.5)	131.2 (129.7-132.7)	137.8 (135.8-139.8)
DBP, mean (95% CI), mm Hg	72.2 (71.5-72.9)	75.2 (74.3-76.2)	71.3 (70.3-72.3)	75.4 (73.8-76.9)

Abbreviations: BMI, body mass index (calculated as weight in kilograms divided by height in meters squared); DBP, diastolic blood pressure; SBP, systolic blood pressure.

### Mean SBP and DBP Using NHANES 2015-2020 Data

Among non-Hispanic Black and non-Hispanic White US adults, the mean SBP was 130.7 (95% CI, 129.0-132.5) and 124.2 (95% CI, 123.1-125.3) mm Hg, respectively (difference, 6.5 [95% CI, 4.5-8.5] mm Hg), for those not taking antihypertensive medication and 137.8 (95% CI, 135.8-139.8) and 131.2 (95% CI, 129.7-132.7) mm Hg, respectively (difference, 6.5 [95% CI, 4.0-9.1] mm Hg) for those taking antihypertensive medication (Table 2). The mean SBP was higher among non-Hispanic Black compared with non-Hispanic White men and women in each age group. The mean DBP by antihypertensive medication status, age, race and ethnicity, and sex is also shown in Table 2.

**Table 2.** Mean SBP and DBP Among Non-Hispanic Black and Non-Hispanic White US Adults by Use of Antihypertensive Medication, National Health and Nutrition Examination Survey 2015-2020 Cycles

Age, y	SBP, mm Hg		Difference in SBP, mm Hg	DBP, mm Hg		Difference in DBP, mm Hg
	Non-Hispanic White adults	Non-Hispanic Black adults		Non-Hispanic White adults	Non-Hispanic Black adults	
**Not taking antihypertensive medication (n = 2018)**						
All						
45-64	122.0 (120.8-123.2)	128.6 (126.8-130.3)	6.5 (4.5-8.6)	73.6 (72.9-74.4)	75.9 (74.8-77.0)	2.2 (1.0-3.5)
65-74	126.7 (124.4-128.9)	137.7 (133.1-142.3)	11.0 (5.5-16.5)	69.5 (67.9-71.2)	75.0 (72.7-77.4)	5.5 (2.8-8.1)
≥75	135.7 (132.9-138.5)	145.0 (138.8-151.2)	9.3 (2.7-15.9)	66.6 (65.2-68.0)	67.1 (63.1-71.1)	0.5 (−3.9-4.9)
Overall	124.2 (123.1-125.3)	130.7 (129.0-132.5)	6.5 (4.5-8.5)	72.2 (71.5-72.9)	75.2 (74.3-76.2)	3.0 (1.8-4.2)
Men						
45-64	124.3 (122.9-125.6)	130.1 (127.9-132.3)	5.8 (3.4-8.3)	75.3 (74.2-76.4)	76.7 (75.3-78.2)	1.4 (−0.1-3.0)
65-74	125.5 (122.7-128.3)	135.5 (130.2-140.9)	10.0 (3.6-16.3)	70.0 (67.8-72.2)	74.6 (71.4-77.9)	4.6 (1.0-8.3)
≥75	132.4 (129.9-134.9)	140.8 (134.2-147.3)	8.3 (1.3-15.4)	65.3 (63.3-67.3)	68.7 (62.5-74.9)	3.5 (−2.9-9.8)
Overall	125.2 (123.9-126.5)	131.6 (129.3-133.9)	6.4 (4.0-8.9)	73.4 (72.4-74.4)	75.9 (74.7-77.0)	2.4 (1.0-3.9)
Women						
45-64	120.1 (118.3-121.8)	127.2 (124.4-130.0)	7.1 (3.6-10.7)	72.2 (71.3-73.1)	75.1 (73.2-77.1)	2.9 (0.8-5.1)
65-74	127.8 (124.2-131.3)	140.5 (133.2-147.8)	12.7 (4.9-20.6)	69.1 (67.0-71.2)	75.5 (72.3-78.7)	6.4 (2.7-10.0)
≥75	137.9 (133.6-142.1)	149.7 (138.0-161.4)	11.9 (−0.1-23.8)	67.5 (65.6-69.5)	65.2 (60.2-70.3)	−2.3 (−7.8-3.2)
Overall	123.4 (121.6-125.1)	129.9 (126.8-132.9)	6.5 (2.9-10.1)	71.1 (70.3-72.0)	74.6 (72.9-76.3)	3.5 (1.6-5.4)
**Taking antihypertensive medication (n = 1711)**						
All						
45-64	126.9 (124.9-128.8)	134.9 (132.8-137.1)	8.1 (5.4-10.7)	74.3 (73.1-75.5)	77.7 (75.8-79.6)	3.4 (0.9-5.9)
65-74	132.8 (130.0-135.7)	138.8 (136.4-141.1)	5.9 (2.3-9.5)	69.7 (67.4-72.0)	72.1 (70.2-74.0)	2.4 (−1.2-6.0)
≥75	139.0 (136.0-142.0)	149.3 (143.4-155.1)	10.3 (4.3-16.2)	66.8 (65.1-68.4)	69.6 (66.2-73.1)	2.9 (−0.9-6.6)
Overall	131.2 (129.7-132.7)	137.8 (135.8-139.8)	6.5 (4.0-9.1)	71.3 (70.3-72.3)	75.4 (73.8-76.9)	4.0 (1.9-6.1)
Men						
45-64	127.7 (125.0-130.4)	137.7 (135.9-139.5)	10.0 (6.5-13.4)	74.5 (72.9-76.1)	79.6 (76.8-82.3)	5.1 (1.6-8.6)
65-74	132.2 (128.3-136.1)	138.1 (133.4-142.9)	5.9 (−0.4-12.2)	70.0 (67.4-72.6)	73.6 (71.6-75.6)	3.6 (0.1-7.1)
≥75	135.8 (133.0-138.7)	141.1 (135.1-147.1)	5.2 (−1.3-11.8)	66.6 (64.5-68.6)	66.8 (63.4-70.2)	0.3 (−3.8-4.4)
Overall	130.5 (128.7-132.3)	138.2 (136.4-139.9)	7.7 (5.2-10.1)	71.8 (70.7-72.8)	76.9 (74.7-79.2)	5.2 (2.5-7.9)
Women						
45-64	126.1 (123.4-128.8)	133.0 (130.0-136.0)	7.0 (3.3-10.6)	74.1 (72.4-75.8)	76.4 (74.6-78.2)	2.3 (−0.4-5.0)
65-74	133.3 (129.6-137.0)	139.1 (136.1-142.2)	5.8 (1.3-10.4)	69.5 (66.3-72.6)	71.2 (68.3-74.1)	1.7 (−3.1-6.5)
≥75	141.0 (137.0-145.0)	152.7 (145.6-159.9)	11.8 (4.5-19.1)	66.9 (64.6-69.1)	70.8 (66.4-75.2)	3.9 (−0.9-8.7)
Overall	131.8 (129.8-133.8)	137.5 (134.9-140.1)	5.7 (2.4-9.0)	70.9 (69.5-72.4)	74.4 (72.7-76.0)	3.4 (1.0-5.8)

Abbreviations: DBP, diastolic blood pressure; SBP, systolic blood pressure.

### Number of US Adults 45 Years and Older, NHANES 2015-2020 Data

In 2015 to 2020 in the US, there were 2.3 (95% CI, 1.9-2.7) million non-Hispanic Black men and 2.4 (95% CI, 1.8-3.1) million non-Hispanic Black women 45 years and older not taking antihypertensive medication. There were 2.1 (95% CI, 1.6-2.5) million non-Hispanic Black men and 3.3 (95% CI, 2.5-4.0) million non-Hispanic Black women 45 years and older taking antihypertensive medication (eTable 1 in Supplement 1).

### Ten-Year Cumulative Incidence of CVD and CVD Mortality Using REGARDS Data

The 10-year cumulative incidence of CVD events was 9.4% (95% CI, 8.5%-10.3%) among non-Hispanic Black adults 45 years and older not taking antihypertensive medication and 17.6% (95% CI, 16.6%-18.6%) among those taking antihypertensive medication (Table 3). The 10-year cumulative incidence of CVD deaths among non-Hispanic Black adults 45 years and older not taking antihypertensive medication was 5.9% (95% CI, 5.1%-6.7%); among those taking antihypertensive medication, it was 10.1% (95% CI, 9.4%-10.8%). For non-Hispanic Black adults not taking and taking antihypertensive medication, the 10-year cumulative incidence of CVD events and CVD mortality was higher among men compared with women and increased with age. The 10-year cumulative incidence of CHD, stroke, and heart failure among non-Hispanic Black men and women are reported in eTable 2 in Supplement 1.

**Table 3.** Ten-Year Cumulative Incidence of CVD Events and Mortality Among Non-Hispanic Black Adults From the Reasons for Geographic and Racial Differences in Stroke Study

Age, y	Ten-y Cumulative incidence, % (95% CI)					
	Not taking antihypertensive medication			Taking antihypertensive medication		
	Overall	Non-Hispanic Black men	Non-Hispanic Black women	Overall	Non-Hispanic Black men	Non-Hispanic Black women
CVD events						
45-64	6.2 (5.1-7.4)	9.0 (7.4-11.1)	4.2 (3.1-5.5)	14.1 (13.0-15.3)	17.1 (15.2-19.2)	12.5 (11.0-14.2)
65-74	11.9 (10.1-14.0)	14.5 (11.9-17.8)	9.6 (7.5-12.3)	20.4 (18.8-22.2)	22.1 (19.8-24.7)	19.5 (17.6-21.5)
≥75	17.7 (14.5-21.6)	20.0 (15.5-26.0)	15.5 (11.7-20.5)	21.9 (19.7-24.3)	23.9 (20.3-28.3)	20.7 (17.9-23.9)
Overall	9.4 (8.5-10.3)	12.3 (10.8-14.0)	7.1 (6.0-8.3)	17.6 (16.6-18.6)	20.1 (18.5-21.8)	16.2 (15.3-17.3)
CVD deaths						
45-64	2.3 (1.7-3.0)	3.5 (2.5-5.0)	1.4 (0.9-2.2)	6.7 (5.9-7.7)	9.6 (8.1-11.5)	5.2 (4.2-6.4)
65-74	7.4 (6.0-9.1)	8.7 (6.4-11.8)	6.3 (4.4-8.9)	10.7 (9.3-12.3)	13.5 (11.4-16.0)	9.1 (7.7-10.7)
≥75	17.7 (14.3-21.9)	20.1 (15.5-26.2)	15.4 (11.8-20.0)	18.3 (16.1-20.8)	21.3 (17.9-25.3)	16.5 (14.0-19.5)
Overall	5.9 (5.1-6.7)	7.6 (6.5-9.0)	4.5 (3.7-5.6)	10.1 (9.4-10.8)	13.1 (11.9-14.4)	8.4 (7.6-9.3)

Abbreviation: CVD, cardiovascular disease.

### Reduction in Incident Events and Deaths With Achievement of Equity in SBP

The RRs of incident CVD, stroke, CHD, and heart failure and CVD mortality associated with a 5–mm Hg reduction in SBP from the BPLTTC and calibrated to reflect the difference in SBP between non-Hispanic Black and non-Hispanic White US adults are shown in eTable 3 in Supplement 1. Achieving equity in SBP between non-Hispanic Black and White adults was projected to reduce the number of CVD events during 10 years by 50 434 (95% CI, 33 985-71 137) among non-Hispanic Black US adults not taking antihypertensive medication and 122 881 (95% CI, 83 220-176 826) among non-Hispanic Black adults taking antihypertensive medication (Table 4). Achieving equity in SBP between non-Hispanic Black and White adults was projected to reduce the number of CVD deaths during 10 years by 21 703 (95% CI, 7313-40 278) among non-Hispanic Black US adults not taking antihypertensive medication and 55 055 (95% CI, 19 823-99 693) among non-Hispanic Black adults taking antihypertensive medication.

**Table 4.** Incident CVD Events and Deaths Expected With Current SBP Levels and With SBP Equity Among Non-Hispanic Black Men and Women

Age, y	Overall events			Men			Women		
	No. in millions (95% CI)		Difference, No. (95% CI)	No. in millions (95% CI)		Difference, No. (95% CI)	No. in millions (95% CI)		Difference, No. (95% CI)
	Current SBP	SBP equity		Current SBP	SBP equity		Current SBP	SBP equity	
**CVD events**									
Antihypertensive medication use									
45-64	0.25 (0.21-0.29)	0.22 (0.18-0.26)	27 759 (17 704-42 052)	0.16 (0.13-0.20)	0.15 (0.12-0.18)	17 030 (8786-36 794)	0.09 (0.07-0.11)	0.07 (0.06-0.10)	10 729 (5713-17 785)
65-74	0.07 (0.06-0.09)	0.06 (0.05-0.07)	13 656 (7696-21 832)	0.05 (0.04-0.06)	0.04 (0.03-0.05)	8380 (3684-15 362)	0.02 (0.02-0.03)	0.02 (0.02-0.03)	5276 (2195-9218)
≥75	0.05 (0.04-0.06)	0.04 (0.04-0.06)	9019 (3391-15 959)	0.03 (0.02-0.04)	0.03 (0.02-0.04)	4658 (1068-9879)	0.02 (0.02-0.03)	0.02 (0.01-0.02)	4361 (90-9237)
Total	0.38 (0.33-0.42)	0.33 (0.28-0.37)	50 434 (33 985-71 137)	0.24 (0.21-0.30)	0.21 (0.18-0.25)	30 068 (19 134-45 102)	0.13 (0.11-0.16)	0.11 (0.09-0.14)	20 366 (12 232-31 022)
Antihypertensive medication nonuse									
45-64	0.49 (0.45-0.53)	0.42 (0.37-0.46)	71 789 (45 183-101 755)	0.24 (0.21-0.27)	0.20 (0.17-0.23)	40 977 (26 220-59 994)	0.25 (0.22-0.29)	0.22 (0.19-0.26)	30 813 (14 016-53 690)
65-74	0.24 (0.22-0.26)	0.21 (0.19-0.24)	25 151 (10 462-43 603)	0.10 (0.09-0.11)	0.09 (0.07-0.10)	10 209 (673-20 959)	0.14 (0.13-0.16)	0.13 (0.11-0.15)	14 943 (3894-30 445)
≥75	0.16 (0.14-0.18)	0.13 (0.16-0.21)	25 941 (11 427-43 981)	0.05 (0.04-0.06)	0.05 (0.04-0.06)	4.909 (−1189-11 992)	0.11 (0.09-0.12)	0.08 (0.07-0.11)	21 032 (7594-36 953)
Total	0.89 (0.84-0.93)	0.76 (0.70-0.82)	122 881 (83 220-176 826)	0.39 (0.36-0.42)	0.33 (0.30-0.36)	56 094 (35 455-82 179)	0.50 (0.47-0.54)	0.43 (0.39-0.48)	66 787 (40 125-104 098)
**CVD deaths**									
Antihypertensive medication use									
45-64	0.09 (0.07-0.12)	0.08 (0.06-0.11)	7960 (2231-12 572)	0.06 (0.04-0.09)	0.06 (0.04-0.09)	5178 (1625-10 970)	0.03 (0.02-0.04)	0.03 (0.02-0.04)	2782 (853-6481)
65-74	0.05 (0.04-0.06)	0.04 (0.03-0.05)	6657 (2170-13 686)	0.03 (0.02-0.04)	0.03 (0.02-0.04)	3947 (1287-9472)	0.02 (0.01-0.02)	0.01 (0.01-0.02)	2711 (783-6438)
≥75	0.05 (0.04-0.06)	0.05 (0.04-0.06)	7085 (1708-15 542)	0.03 (0.02-0.04)	0.03 (0.02-0.04)	3662 (599-9651)	0.02 (0.02-0.03)	0.02 (0.01-0.03)	3423 (164-9334)
Total	0.19 (0.16-0.22)	0.17 (0.14-0.20)	21 703 (7313-40 278)	0.13 (0.10-0.15)	0.11 (0.09-0.14)	12 787 (4352-24 754)	0.07 (0.05-0.08)	0.06 (0.04-0.07)	8916 (2849-18 245)
Antihypertensive medication nonuse									
45-64	0.24 (0.21-0.27)	0.21 (0.18-0.25)	28 102 (9696-51 558)	0.13 (0.11-0.16)	0.12 (0.09-0.14)	18 123 (6185-33 283)	0.10 (0.08-0.13)	0.09 (0.07-0.12)	9979 (3125-23 432)
65-74	0.13 (0.11-0.14)	0.12 (0.10-0.13)	10 291 (3220-23 863)	0.06 (0.05-0.07)	0.05 (0.04-0.07)	4859 (613-13 171)	0.07 (0.06-0.08)	0.06 (0.05-0.07)	5432 (1292-14 664)
≥75	0.13 (0.12-0.15)	0.11 (0.09-0.13)	16 662 (5229-37 515)	0.05 (0.04-0.06)	0.04 (0.03-0.05)	3401 (−336-10 681)	0.08 (0.07-0.10)	0.07 (0.05-0.09)	13 262 (3559-28 870)
Total	0.50 (0.46-0.53)	0.44 (0.39-0.49)	55 055 (19 823-99 693)	0.24 (0.21-0.27)	0.21 (0.18-0.24)	26 382 (9373-49 031)	0.26 (0.23-0.29)	0.23 (0.19-0.26)	28 672 (10 152-57 705)

Abbreviations: CVD, cardiovascular disease; SBP, systolic blood pressure.

The largest proportion of CVD events among non-Hispanic Black adults not taking and taking antihypertensive medications were prevented among those aged 45 to 64 years (55% and 58%, respectively) (Figure). An estimated 37% and 51% of CVD deaths prevented would occur among non-Hispanic Black adults aged 45 to 64 years not taking and taking antihypertensive medication, respectively (eFigure 3 in Supplement 1).

**Figure.**
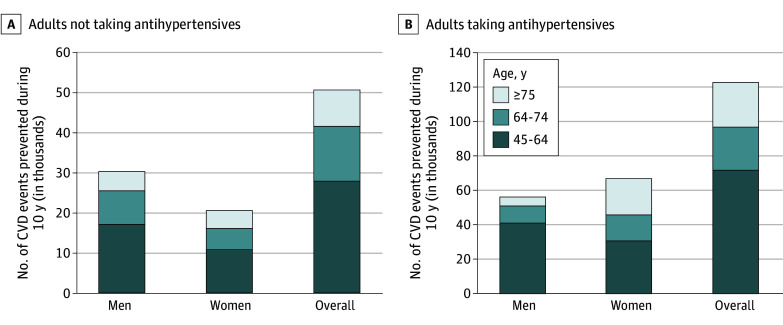
Preventable Cardiovascular Disease (CVD) Events Among Non-Hispanic Black Adults by Achieving Systolic Blood Pressure Equity With White Adults Data are stratified between those not taking (A) and taking (B) antihypertensive medication by sex and age.

The estimated number of CVD events among non-Hispanic Black adults prevented by achieving SBP equity between non-Hispanic Black and White adults for CHD, stroke, and heart failure are shown in eTables 4 to 6 in Supplement 1. Smaller and larger effectiveness of the intervention in the US population compared with the BPLTTC are shown in eTables 7 to 16 in Supplement 1.

## Discussion

In this nationally representative modeling study, mean SBP was higher among non-Hispanic Black compared with White US adults not taking and taking antihypertensive medication. Our findings suggest that if SBP among non-Hispanic Black adults was the same as among non-Hispanic White adults, more than 170 000 CVD events and 75 000 CVD deaths among non-Hispanic Black adults could be prevented. More than half of the CVD events could be prevented among Black adults 45 to 64 years of age.

The risk for CVD increases with higher SBP, even within the range considered normal by current guidelines.^5^ Consistent with prior studies, SBP was higher among non-Hispanic Black US adults compared with non-Hispanic White US adults not taking antihypertensive medication.^7^ The substantial number of CVD events and deaths that could be prevented among non-Hispanic Black adults not taking antihypertensive medication if equity in SBP was achieved emphasizes the value of the prevention of hypertension. Randomized clinical trials have demonstrated that physical activity, adherence to the Dietary Approaches to Stop Hypertension diet, weight loss, reduction in dietary sodium levels, and increased dietary potassium levels lower BP among adults with and without hypertension.^4,5,18^ Few studies have translated results from these trials into implementable community-based hypertension prevention programs or tailored interventions for Black communities.^19,20^ Current initiatives, including the American Heart Association RESTORE (Addressing Social Determinants to Prevent Hypertension) network,^21^ are testing approaches to increase equity in hypertension prevention in Black communities by addressing social determinants of health and meeting individuals where they work, pray, and live.

Analyses of NHANES data have shown that BP control among US adults with hypertension has declined during the past decade, with a consistently lower percentage of non-Hispanic Black adults achieving controlled BP compared with non-Hispanic White adults.^22,23^ In the present study, achieving the same BP level for non-Hispanic Black and non-Hispanic White adults would result in the mean SBP being close to the 2025 American College of Cardiology–American Heart Association BP guideline goal among those taking antihypertensive medication, thus improving control rates. The large number of incident CVD events and deaths that could be prevented by achieving SBP equity and controlling SBP identified in the present study highlights a substantial opportunity for BP control initiatives to increase equity in health. Health systems have implemented quality improvement programs using the Kaiser Permanente Northern California model.^24^ These interventions centered around team-based care led by community health workers have achieved high levels of BP control for both Black and White patients and could be more widely implemented to increase equity in BP.^25^

Previous studies have shown that 45% to 60% of health disparities are attributable to socioeconomic, behavioral, and environmental determinants.^26,27^ Interventions and policies that address social and structural root causes of disparities in BP have a substantial opportunity to create BP equity. Developing interventions with the goal of reducing the impact of structural racism and social determinants of health on BP control and connecting patients with community programs and resources that address social determinants of health could aid in increasing equity in BP. Furthermore, changing policies and laws that perpetuate poverty and limit economic opportunity in Black communities may have a large impact on achieving equity by targeting upstream root causes rather than focusing on individual- or even community-level interventions.

Our findings suggest that approximately half the estimated incident CVD events and deaths that could be prevented by achieving equity in SBP would occur among non-Hispanic Black adults aged 45 to 64 years. These results suggest that public health interventions and policies to improve disparities in adverse BP outcomes may benefit from focusing on healthy aging across the life course. Hypertension develops at younger ages among non-Hispanic Black compared with non-Hispanic White populations and disparities in BP emerge during adolescence.^28,29^ Prior studies suggest that engaging in guideline-recommended physical activity and adhering to the Dietary Approaches to Stop Hypertension diet lowers BP across all age groups.^30,31^ Extending lifestyle education on health behaviors traditionally provided by primary care clinicians and dieticians to other advocates such as community health workers could increase reach and awareness of recommended lifestyle behavior change to younger populations.^32^

### Strengths and Limitations

Strengths of this study include the use of nationally representative data from NHANES, which provides estimates of mean SBP, assessed following a rigorous protocol. Additionally, the large sample of Black adults from across the US in the REGARDS study provided reliable estimates of CVD incidence and mortality rates following a rigorous adjudication process.

Our findings should be considered in the context of several limitations. Each participant’s BP was measured at a single study visit, and guidelines recommend obtaining a mean using multiple BP measurements obtained during 2 or more visits.^5^ Although inequities in BP between non-Hispanic Black and non-Hispanic White populations arise at early ages, the present study was restricted to those 45 years or older due to the REGARDS study not including children or young adults. However, CVD incidence and mortality rates are very low among those younger than 45 years.^33^ This analysis assumed that RR estimates from the BPLTTC are applicable to the general US population represented in NHANES and to the REGARDS study cohort. Differences in demographic, socioeconomic, and clinical characteristics across these data sources could influence the magnitude of observed associations and may limit the transportability of trial-based estimates. However, the RR in the BPLLTC has been shown to be remarkably consistent across levels of predicted risk, for women and men and for age groups.^34,35,36^ In addition, the BPLTTC includes 51 trials, many conducted in the US, ensuring a high degree of generalizability. While the present study quantifies the benefits of achieving equity in SBP on incident CVD events and deaths, the advantages would likely extend to increasing equity in life expectancy, health care costs, and morbidity in the US.^37^

## Conclusions

In this modeling study, achieving SBP equity between non-Hispanic Black and non-Hispanic White US adults was estimated to prevent more than 170 000 incident CVD events and 75 000 CVD deaths during the next decade among non-Hispanic Black US adults. Our findings suggest that approximately half of these CVD events and deaths could be prevented among non-Hispanic Black adults aged 45 to 64 years. The large number of incident CVD events and deaths that could be prevented among middle-aged Black adults suggests that initiatives to maintain normal BP among those without hypertension and achieve BP control for those with hypertension could have a substantial impact on health equity in the US.

## Notes

Supplement 1. **eTable 1.** Number in Millions of Non-Hispanic Black US Adults, NHANES 2015-2020 Cycles **eTable 2.** Ten-Year Cumulative Incidence of Coronary Heart Disease, Stroke, and Heart Failure Among Non-Hispanic Black Men and Women, REGARDS Study **eTable 3.** Relative Risk for Outcomes Associated With a 5–mm Hg Reduction in Systolic Blood Pressure From Randomized Trials in the Blood Pressure Lowering Treatment Trialists Collaboration Among Those Without a History of Cardiovascular Disease and Calibrated Relative Risk Representing the Difference in Systolic Blood Pressure Between Non-Hispanic Black and White Adults Taking and Not Taking Antihypertensive Medication **eTable 4.** Number of Incident Coronary Heart Disease Events Expected With Current SBP Levels and With SBP Equity Among Non-Hispanic Black Adults **eTable 5.** Number of Incident Stroke Events Expected With Current SBP Levels and With SBP Equity Among Non-Hispanic Black Adults **eTable 6.** Number of Incident Heart Failure Events Expected With Current SBP Levels and With SBP Equity Among Non-Hispanic Black Adults **eTable 7.** Number of Cardiovascular Disease Events Expected With SBP Equity Accounting for a 10% Decrease in Effectiveness of the Intervention Reported From the Blood Pressure–Lowering Treatment Trialists Collaboration **eTable 8.** Number of Coronary Heart Disease Events Expected With SBP Equity Accounting for a 10% Decrease in Effectiveness of the Intervention Reported From the Blood Pressure–Lowering Treatment Trialists Collaboration **eTable 9.** Number of Stroke Events Expected With SBP Equity Accounting for a 10% Decrease in Effectiveness of the Intervention Reported From the Blood Pressure–Lowering Treatment Trialists Collaboration **eTable 10.** Number of Heart Failure Events Expected With SBP Equity Accounting for a 10% Decrease in Effectiveness of the Intervention Reported From the Blood Pressure–Lowering Treatment Trialists Collaboration **eTable 11.** Number of Cardiovascular Mortality Events Expected With SBP Equity Accounting for a 10% Decrease in Effectiveness of the Intervention Reported From the Blood Pressure–Lowering Treatment Trialists Collaboration **eTable 12.** Number of Cardiovascular Disease Events Expected With SBP Equity Accounting for a 10% Increase in Effectiveness of the Intervention Reported From the Blood Pressure–Lowering Treatment Trialists Collaboration **eTable 13.** Number of Coronary Heart Disease Events Expected With SBP Equity Accounting for a 10% Increase in Effectiveness of the Intervention Reported From the Blood Pressure–Lowering Treatment Trialists Collaboration **eTable 14.** Number of Stroke Events Expected With SBP Equity Accounting for a 10% Increase in Effectiveness of the Intervention Reported From the Blood Pressure–Lowering Treatment Trialists Collaboration **eTable 15.** Number of Heart Failure Events Expected With SBP Equity Accounting for a 10% Increase in Effectiveness of the Intervention Reported From the Blood Pressure–Lowering Treatment Trialists Collaboration **eTable 16.** Number of Cardiovascular Mortality Events Expected With SBP Equity Accounting for a 10% Increase in Effectiveness of the Intervention Reported From the Blood Pressure–Lowering Treatment Trialists Collaboration **eFigure 1.** NHANES Study Inclusion Criteria **eFigure 2.** REGARDS Study Inclusion Criteria **eFigure 3.** Number of Cardiovascular Disease Deaths That Could Be Prevented Among Non-Hispanic Black Adults With Systolic Blood Pressure Equity Between Non-Hispanic Black and White Adults Not Taking and Taking Antihypertensive Medication by Sex and Age

Supplement 2.
Data Sharing Statement
