# Supplementary material for: Prevention of Cardiovascular Disease Events and Deaths Among Black Adults Via Systolic Blood Pressure Equity
Source: JAMA Netw Open. 2025 Nov 4;8(11):e2541336. doi: 10.1001/jamanetworkopen.2025.41336 (PMC12587195; doi:10.1001/jamanetworkopen.2025.41336)
Supplement: Supplement 1. — eTable 1. Number in Millions of Non-Hispanic Black US Adults, NHANES 2015-2020 Cycles eTable 2. Ten-Year Cumulative Incidence of Coronary Heart Disease, Stroke, and Heart Failure Among Non-Hispanic Black Men and Women, REGARDS Study eTable 3. Relative Risk for Outcomes Associated With a 5–mm Hg Reduction in Systolic Blood Pressure From Randomized Trials in the Blood Pressure Lowering Treatment Trialists Collaboration Among Those Without a History of Cardiovascular Disease and Calibrated Relative Risk Representing the Difference in Systolic Blood Pressure Between Non-Hispanic Black and White Adults Taking and Not Taking Antihypertensive Medication eTable 4. Number of Incident Coronary Heart Disease Events Expected With Current SBP Levels and With SBP Equity Among Non-Hispanic Black Adults eTable 5. Number of Incident Stroke Events Expected With Current SBP Levels and With SBP Equity Among Non-Hispanic Black Adults eTable 6. Number of Incident Heart Failure Events Expected With Current SBP Levels and With SBP Equity Among Non-Hispanic Black Adults eTable 7. Number of Cardiovascular Disease Events Expected With SBP Equity Accounting for a 10% Decrease in Effectiveness of the Intervention Reported From the Blood Pressure–Lowering Treatment Trialists Collaboration eTable 8. Number of Coronary Heart Disease Events Expected With SBP Equity Accounting for a 10% Decrease in Effectiveness of the Intervention Reported From the Blood Pressure–Lowering Treatment Trialists Collaboration eTable 9. Number of Stroke Events Expected With SBP Equity Accounting for a 10% Decrease in Effectiveness of the Intervention Reported From the Blood Pressure–Lowering Treatment Trialists Collaboration eTable 10. Number of Heart Failure Events Expected With SBP Equity Accounting for a 10% Decrease in Effectiveness of the Intervention Reported From the Blood Pressure–Lowering Treatment Trialists Collaboration eTable 11. Number of Cardiovascular Mortality Events Expected With SBP Equity Accounting f [file jamanetwopen-e2541336-s001.pdf]

Supplementary Online Content

Hardy ST, Huang L, Coantonio LD, et al. Prevention of cardiovascular disease events and deaths among Black adults via blood pressure equity. *JAMA Netw Open.* 2025;8(11):e2541336. doi:10.1001/jamanetworkopen.2025.41336

eTable 1. Number in Millions of Non-Hispanic Black US Adults, NHANES 2015-2020 Cycles

eTable 2. Ten-Year Cumulative Incidence of Coronary Heart Disease, Stroke, and Heart Failure Among Non-Hispanic Black Men and Women, REGARDS Study

eTable 3. Relative Risk for Outcomes Associated With a 5–mm Hg Reduction in Systolic Blood Pressure From Randomized Trials in the Blood Pressure Lowering Treatment Trialists Collaboration Among Those Without a History of Cardiovascular Disease and Calibrated Relative Risk Representing the Difference in Systolic Blood Pressure Between Non-Hispanic Black and White Adults Taking and Not Taking Antihypertensive Medication

eTable 4. Number of Incident Coronary Heart Disease Events Expected With Current SBP Levels and With SBP Equity Among Non-Hispanic Black Adults

eTable 5. Number of Incident Stroke Events Expected With Current SBP Levels and With SBP Equity Among Non-Hispanic Black Adults

eTable 6. Number of Incident Heart Failure Events Expected With Current SBP Levels and With SBP Equity Among Non-Hispanic Black Adults

eTable 7. Number of Cardiovascular Disease Events Expected With SBP Equity Accounting for a 10% Decrease in Effectiveness of the Intervention Reported From the Blood Pressure–Lowering Treatment Trialists Collaboration

eTable 8. Number of Coronary Heart Disease Events Expected With SBP Equity Accounting for a 10% Decrease in Effectiveness of the Intervention Reported From the Blood Pressure–Lowering Treatment Trialists Collaboration

eTable 9. Number of Stroke Events Expected With SBP Equity Accounting for a 10% Decrease in Effectiveness of the Intervention Reported From the Blood Pressure–Lowering Treatment Trialists Collaboration

eTable 10. Number of Heart Failure Events Expected With SBP Equity Accounting for a 10% Decrease in Effectiveness of the Intervention Reported From the Blood Pressure–Lowering Treatment Trialists Collaboration

eTable 11. Number of Cardiovascular Mortality Events Expected With SBP Equity Accounting for a 10% Decrease in Effectiveness of the Intervention Reported From the Blood Pressure–Lowering Treatment Trialists Collaboration

eTable 12. Number of Cardiovascular Disease Events Expected With SBP Equity Accounting for a 10% Increase in Effectiveness of the Intervention Reported From the Blood Pressure–Lowering Treatment Trialists Collaboration

eTable 13. Number of Coronary Heart Disease Events Expected With SBP Equity Accounting for a 10% Increase in Effectiveness of the Intervention Reported From the Blood Pressure–Lowering Treatment Trialists Collaboration

eTable 14. Number of Stroke Events Expected With SBP Equity Accounting for a 10% Increase in Effectiveness of the Intervention Reported From the Blood Pressure–Lowering Treatment Trialists Collaboration

eTable 15. Number of Heart Failure Events Expected With SBP Equity Accounting for a 10% Increase in Effectiveness of the Intervention Reported From the Blood Pressure–Lowering Treatment Trialists Collaboration

© 2025 Hardy ST et al. *JAMA Network Open*.

eTable 16. Number of Cardiovascular Mortality Events Expected With SBP Equity Accounting for a 10% Increase in Effectiveness of the Intervention Reported From the Blood Pressure–Lowering Treatment Trialists Collaboration

eFigure 1. NHANES Study Inclusion Criteria

eFigure 2. REGARDS Study Inclusion Criteria

eFigure 3. Number of Cardiovascular Disease Deaths That Could Be Prevented Among Non-Hispanic Black Adults With Systolic Blood Pressure Equity Between Non-Hispanic Black and White Adults Not Taking and Taking Antihypertensive Medication by Sex and Age

This supplementary material has been provided by the authors to give readers additional information about their work.

© 2025 Hardy ST et al. *JAMA Network Open*.

eTable 1. Number in Millions of Non-Hispanic Black US Adults, NHANES 2015-2020 Cycles

	Not taking antihypertensive medication			Taking antihypertensive medication		
**Age, years**	**Overall**	**Non-Hispanic Black Men**	**Non-Hispanic Black Women**	**Overall**	**Non-Hispanic Black Men**	**Non-Hispanic Black Women**
**NHANES Data**						
**Number of non-Hispanic Black US adults (95% CI) in millions**						
45 – 64	3.9 (3.1, 4.7)	1.8 (1.5, 2.1)	2.0 (1.5, 2.6)	3.4 (2.7, 4.2)	1.4 (1.1, 1.7)	2.0 (1.5, 2.5)
65 – 74	0.6 (0.4, 0.8)	0.3 (0.2, 0.4)	0.3 (0.2, 0.4)	1.2 (0.9, 1.5)	0.4 (0.3, 0.6)	0.7 (0.5, 1.0)
≥75	0.3 (0.2, 0.4)	0.2 (0.1, 0.2)	0.1 (0.1, 0.2)	0.7 (0.5, 0.9)	0.2 (0.1, 0.3)	0.5 (0.3, 0.7)
Total	4.8 (3.8 - 5.7)	2.3 (1.9, 2.7)	2.4 (1.8, 3.1)	5.3 (4.1, 6.5)	2.1 (1.6, 2.5)	3.3 (2.5, 4.0)

© 2025 Hardy ST et al. *JAMA Network Open*.

eTable 2. Ten-Year Cumulative Incidence of Coronary Heart Disease, Stroke, and Heart Failure Among Non-Hispanic Black Men and Women, REGARDS Study

	**Non-Hispanic Black adults not taking antihypertensive medication**			**Non-Hispanic Black adults taking antihypertensive medication**		
Age, years	Overall	Men	Women	Overall	Men	Women
	Coronary Heart Disease					
45 – 64	3.6 (2.7, 4.8)	5.3 (4.0, 7.1)	2.4 (1.6, 3.5)	7.7 (6.8, 8.7)	10.2 (8.7, 11.9)	6.4 (5.5, 7.5)
65 – 74	6.6 (5.2, 8.2)	9.3 (6.8, 12.6)	4.2 (2.9, 6.1)	11.6 (10.4, 13.0)	14.6 (12.4, 17.2)	9.8 (8.5, 11.4)
≥75	10.0 (7.8, 12.8)	13.1 (9.5, 18.0)	7.1 (4.6, 11.1)	11.0 (9.5, 12.7)	12.2 (9.7, 15.3)	10.2 (8.1, 12.9)
Overall	5.3 (4.7, 6.1)	7.7 (6.4, 9.2)	3.5 (2.8, 4.4)	9.6 (9.0, 10.2)	12.1 (11.0, 13.3)	8.2 (7.4, 9.1)
	Stroke					
45 – 64	2.1 (1.5, 3.0)	3.2 (2.3, 4.4)	1.4 (0.8, 2.3)	4.1 (3.5, 4.8)	4.6 (3.5, 6.0)	3.9 (3.2, 4.7)
65 – 74	4.1 (3.1, 5.4)	4.0 (2.6, 6.3)	4.1 (2.7, 6.3)	6.6 (5.7, 7.7)	5.8 (4.4, 7.6)	7.1 (5.9, 8.6)
≥75	5.8 (4.0, 8.3)	5.7 (3.2, 9.9)	5.9 (4.0, 8.9)	7.4 (6.2, 8.8)	7.0 (5.0, 9.9)	7.6 (5.9, 9.8)
Overall	3.2 (2.7, 3.8)	3.8 (2.9, 4.9)	2.7 (2.1, 3.6)	5.5 (5.1, 6.0)	5.4 (4.6, 6.4)	5.6 (4.9, 6.4)
	Heart Failure					
45 – 64	1.5 (1.1, 2.0)	1.7 (1.0, 3.0)	1.3 (0.8, 2.2)	5.6 (4.9, 6.3)	7.0 (5.6, 8.6)	4.9 (4.0, 5.9)
65 – 74	3.3 (2.4, 4.4)	4.4 (3.0, 6.6)	2.2 (1.3, 3.8)	7.7 (6.6, 8.9)	8.0 (6.4, 10.0)	7.5 (6.3, 8.9)
≥75	5.2 (3.5, 7.6)	6.5 (3.8, 11.2)	3.9 (2.2, 6.9)	8.4 (6.9, 10.2)	9.4 (7.1, 12.4)	7.8 (6.1, 9.9)
Overall	2.5 (2.1, 2.9)	3.2 (2.4, 4.2)	1.9 (1.4, 2.6)	6.8 (6.2, 7.4)	7.8 (6.8, 8.8)	6.2 (5.6, 6.9)

Data in Table are 10-year cumulative incidence (95% confidence interval).

© 2025 Hardy ST et al. *JAMA Network Open*.

eTable 3. Relative Risk for Outcomes Associated With a 5–mm Hg Reduction in Systolic Blood Pressure From Randomized Trials in the Blood Pressure Lowering Treatment Trialists Collaboration Among Those Without a History of Cardiovascular Disease and Calibrated Relative Risk Representing the Difference in Systolic Blood Pressure Between Non-Hispanic Black and White Adults Taking and Not Taking Antihypertensive Medication

		**Calibrated Relative Risk (95% CI) – representing difference in SBP between NH Black and White adults**	
**Outcome**	**BPLTTC Relative Risk (95% CI) for 5 mm Hg reduction**	**Not taking antihypertensive medication**	**Taking antihypertensive medication**
Cardiovascular disease	0.91 (0.89, 0.94)	0.88 (0.85, 0.92)	0.88 (0.84, 0.93)
Stroke	0.85 (0.80, 0.90)	0.81 (0.76, 0.86)	0.81 (0.74, 0.88)
Coronary heart disease	0.95 (0.91, 0.99)	0.94 (0.92, 0.95)	0.94 (0.91, 0.96)
Heart failure	0.83 (0.77, 0.89)	0.78 (0.73, 0.84)	0.78 (0.71, 0.86)
Cardiovascular disease mortality	0.93 (0.88, 0.98)	0.91 (0.88, 0.94)	0.91 (0.88, 0.94)

BPLTTC - Blood Pressure Lowering Treatment Trialists Collaboration, CI – confidence interval, SBP – systolic blood pressure

Includes those without a history of cardiovascular disease and calibrated relative risk representing the difference in systolic blood pressure between non-Hispanic Black and White adults taking and not taking antihypertensive medication.

© 2025 Hardy ST et al. *JAMA Network Open*.

eTable 4. Number of Incident Coronary Heart Disease Events Expected With Current SBP Levels and With SBP Equity Among Non-Hispanic Black Adults

	Non-Hispanic Black adults (95% confidence interval)								
	**Not taking antihypertensive medication**								
**CHD**	**Overall**			**Men**			**Women**		
**Age, years**	**Number, in millions, of events with current SBP**	**Number, in millions, of events with SBP equity**	**Difference in number of events with current SBP and SBP equity**	**Number, in millions, of events with current SBP**	**Number, in millions, of events with SBP equity**	**Difference in number of events with current SBP and SBP equity**	**Number, in millions, of events with current SBP**	**Number, in millions, of events with SBP equity**	**Difference in number of events with current SBP and SBP equity**
45 – 64	0.15 (0.12, 0.18)	0.14 (0.11, 0.17)	9,067 (2,291, 18,195)	0.10 (0.07, 0.13)	0.09 (0.07, 0.12)	5,598 (1,342, 12,171)	0.05 (0.03, 0.07)	0.05 (0.03, 0.07)	3,469 (930, 8,120)
65 – 74	0.04 (0.03, 0.05)	0.04 (0.03, 0.05)	4,361 (1,145, 9,800)	0.03 (0.02, 0.04)	0.03 (0.02, 0.04)	3,033 (806, 7,533)	0.01 (0.01, 0.02)	0.01 (0.01, 0.01)	1,327 (325, 3,416)
>=75	0.03 (0.02, 0.04)	0.03 (0.02, 0.04)	2,859 (577, 6,945)	0.02 (0.02, 0.03)	0.02 (0.01, 0.03)	1,709 (270, 4,988)	0.01 (0.01, 0.02)	0.01 (0.01, 0.01)	1,150 (47, 3,657)
Total	0.22 (0.18, 0.26)	0.20 (0.17, 0.24)	16,287 (3,831, 31,005)	0.15 (0.12, 0.18)	0.14 (0.11, 0.17)	10,340 (2,591, 20.991)	0.07 (0.05, 0.09)	0.06 (0.05, 0.08)	5,947 (1,567, 12,501)
	**Taking antihypertensive medication**								
**CHD**	**Overall**			**Men**			**Women**		
**Age, years**	**Number, in millions, of events with current SBP**	**Number, in millions, of events with SBP equity**	**Difference in number of events with current SBP and SBP equity**	**Number, in millions, of events with current SBP**	**Number, in millions, of events with SBP equity**	**Difference in number of events with current SBP and SBP equity**	**Number, in millions, of events with current SBP**	**Number, in millions, of events with SBP equity**	**Difference in number of events with current SBP and SBP equity**
45 – 64	0.27 (0.24, 0.30)	0.25 (0.21, 0.28)	22,648 (6,304, 43,721)	0.14 (0.12, 0.17)	0.13 (0.11, 0.15)	13,815 (3,826, 26,100)	0.13 (0.11, 0.15)	0.13 (0.11, 0.15)	8,833 (2,454, 22,016)
65 – 74	0.14 (0.12, 0.15)	0.13 (0.11, 0.14)	7,972 (2,289, 20,131)	0.06 (0.05, 0.07)	0.06 (0.05, 0.07)	3758 (489, 10,813)	0.07 (0.06, 0.08)	0.06 (0.05, 0.07)	4,214 (925, 12,019)
>=75	0.08 (0.07, 0.09)	0.07 (0.06, 0.09)	7,330 (1,969, 17,622)	0.03 (0.02, 0.03)	0.03 (0.02, 0.03)	1,391 (-133, 4,714)	0.05 (0.04, 0.07)	0.03 (0.02, 0.03)	5,938 (1,455, 14,071)
Total	0.49 (0.45, 0.52)	0.45 (0.40, 0.49)	37,950 (11,156, 73,244)	0.23 (0.21, 0.26)	0.21 (0.19, 0.24)	18,965 (5,364, 36,714)	0.25 (0.23, 0.28)	0.21 (0.19, 0.24)	18,985 (5,653, 42,645)

Data in the Table are number of incident CHD events prevented among non-Hispanic Black adults (95% confidence interval). with current systolic blood pressure levels, with systolic blood pressure equity and the difference between these numbers among men and women not taking antihypertensive medication (top panel) and taking antihypertensive medication (bottom panel).

CHD – coronary heart disease, SBP – systolic blood pressure

© 2025 Hardy ST et al. *JAMA Network Open*.

eTable 5. Number of Incident Stroke Events Expected With Current SBP Levels and With SBP Equity Among Non-Hispanic Black Adults

	Non-Hispanic Black adults (95% confidence interval)								
	Not taking antihypertensive medication								
**Stroke**	**Overall**			**Men**			**Women**		
**Age, years**	**Number, in millions, of events with current SBP**	**Number, in millions, of events with SBP equity**	**Difference in number of events with current SBP and SBP equity**	**Number, in millions, of events with current SBP**	**Number, in millions, of events with SBP equity**	**Difference in number of events with current SBP and SBP equity**	**Number, in millions, of events with current SBP**	**Number, in millions, of events with SBP equity**	**Difference in number of events with current SBP and SBP equity**
45 – 64	0.09 (0.06, 0.11)	0.07 (0.05, 0.07)	15,725 (9,084, 25,335)	0.06 (0.04, 0.08)	0.05 (0.03, 0.07)	9,897 (5,012, 17,072)	0.03 (0.02, 0.04)	0.02 (0.01, 0.04)	5,829 (2,857, 11,297)
65 – 74	0.02 (0.02, 0.03)	0.02 (0.01, 0.02)	7,331 (3,914, 12,412)	0.01 (0.01, 0.02)	0.01 (0.01, 0.02)	3,763 (1,702, 7,682)	0.01 (0.01, 0.02)	0.01 (0.01, 0.01)	3,569 (1,530, 7,272)
>=75	0.02 (0.01, 0.02)	0.01 (0.01, 0.02)	4,820 (1,591, 8,839)	0.01 (0.01, 0.02)	0.01 (0.00, 0.01)	2,143 (495, 5,386)	0.01 (0.01, 0.01)	0.01 (0.00, 0.01)	2,677 (108, 5,644)
Total	0.13 (0.10, 0.16)	0.10 (0.08, 0.12)	27,877 (17,242, 41,114)	0.08 (0.06, 0.10)	0.06 (0.05, 0.08)	15,803 (9,162, 25,659)	0.05 (0.03, 0.06)	0.03 (0.02, 0.05)	12,074 (6,662, 19,990)
	Taking antihypertensive medication								
**Stroke**	**Overall**			**Men**			**Women**		
**Age, years**	**Number, in millions, of events with current SBP**	**Number, in millions, of events with SBP equity**	**Difference in number of events with current SBP and SBP equity**	**Number, in millions, of events with current SBP**	**Number, in millions, of events with SBP equity**	**Difference in number of events with current SBP and SBP equity**	**Number, in millions, of events with current SBP**	**Number, in millions, of events with SBP equity**	**Difference in number of events with current SBP and SBP equity**
45 – 64	0.14 (0.12, 0.17)	0.11 (0.09, 0.13)	33,449 (19,165, 50,683)	0.06 (0.05, 0.09)	0.05 (0.03, 0.06)	17,681 (10,624-28,362)	0.08 (0.06 ,0.09)	0.06 (0.05, 0.08)	15,768 (7,100-28,241)
65 – 74	0.08 (0.07, 0.09)	0.06 (0.05, 0.08)	13,504 (5,493, 23,623)	0.03 (0.02, 0.03)	0.02 (0.02, 0.03)	4,446 (438-9,683)	0.05 (0.04 ,0.06)	0.04 (0.03, 0.06)	9,057 (2,625-19,482)
>=75	0.05 (0.04, 0.07)	0.04 (0.03, 0.05)	14,727 (6,399, 25,876)	0.02 (0.01, 0.02)	0.01 (0.01, 0.02)	2,391 (-461, 6,041)	0.04 (0.03, 0.05)	0.03 (0.02, 0.04)	12,337 (4,465, 21,684)
Total	0.27 (0.25, 0.30)	0.21 (0.18, 0.24)	61,680 (39,077, 92,435)	0.10 (0.08, 0.13)	0.08 (0.06, 0.10)	24,518 (14,338, 38,255)	0.17 (0.15, 0.19)	0.13 (0.11, 0.15)	37,162 (21,240, 59,281)

Data in the Table are the number of incident stroke events prevented among non-Hispanic Black adults (95% confidence interval) with current systolic blood pressure levels, with systolic blood pressure equity and the difference between these numbers among men and women not taking antihypertensive medication (top panel) and taking antihypertensive medication (bottom panel)..

SBP – systolic blood pressure

© 2025 Hardy ST et al. *JAMA Network Open*.

eTable 6. Number of Incident Heart Failure Events Expected With Current SBP Levels and With SBP Equity Among Non-Hispanic Black Adults

	Non-Hispanic Black adults (95% confidence interval)								
	**Not taking antihypertensive medication**								
**Heart Failure**	**Overall**			**Men**			**Women**		
**Age, years**	**Number, in millions, of events with current SBP**	**Number, in millions, of events with SBP equity**	**Difference in number of events with current SBP and SBP equity**	**Number, in millions, of events with current SBP**	**Number, in millions, of events with SBP equity**	**Difference in number of events with current SBP and SBP equity**	**Number, in millions, of events with current SBP**	**Number, in millions, of events with SBP equity**	**Difference in number of events with current SBP and SBP equity**
45 – 64	0.06 (0.04, 0.08)	0.05 (0.03, 0.08)	12,250 (6,644, 21,119)	0.03 (0.02, 0.05)	0.02 (0.01, 0.05)	5,949 (2,633, 11,966)	0.03 (0.02, 0.04)	0.02 (0.01, 0.03)	6,302 (3,040.1, 1231)
65 – 74	0.02 (0.02, 0.03)	0.02 (0.01, 0.03)	6,785 (3,577, 12,081)	0.01 (0.01, 0.02)	0.01 (0.01, 0.02)	4,613 (2,184, 9,180)	0.01 (0.00, 0.01)	0.00 (0.00, 0.01)	2,172 (897, 4,882)
>=75	0.02 (0.01, 0.02)	0.01 (0.01, 0.02)	4,742 (1,742, 9,220)	0.01 (0.01, 0.02)	0.01 (0.01, 0.02)	2,775 (653, 6,826)	0.01 (0.00, 0.01)	0.00 (0.00, 0.01)	1,966 (191, 4,720)
Total	0.09 (0.07, 0.12)	0.08 (0.06, 0.11)	23,777 (13,825, 35,947)	0.06 (0.04, 0.08)	0.04 (0.03, 0.06)	13,337 (7,285, 22,773)	0.04 (0.03, 0.05)	0.03 (0.02, 0.04)	10,440 (5,722, 18,203)
	**Taking antihypertensive medication**								
**Heart Failure**	**Overall**			**Men**			**Women**		
**Age, years**	**Number, in millions, of events with current SBP**	**Number, in millions, of events with SBP equity**	**Difference in number of events with current SBP and SBP equity**	**Number, in millions, of events with current SBP**	**Number, in millions, of events with SBP equity**	**Difference in number of events with current SBP and SBP equity**	**Number, in millions, of events with current SBP**	**Number, in millions, of events with SBP equity**	**Difference in number of events with current SBP and SBP equity**
45 – 64	0.19 (0.17, 0.22)	0.14 (0.11, 0.17)	52,318 (31,166, 77,358)	0.10 (0.08, 0.12)	0.07 (0.05, 0.09)	30,153 (18,248, 45,987)	0.10 (0.08, 0.12)	0.07 (0.06, 0.10)	22,165 (9,899, 39,812)
65 – 74	0.09 (0.08, 0.10)	0.07 (0.06, 0.09)	17,722 (7,549, 31,211)	0.03 (0.03, 0.04)	0.03 (0.02, 0.04)	6,921 (675, 14,650)	0.06 (0.05, 0.07)	0.04 (0.03, 0.06)	10,801 (3,089, 22,501)
>=75	0.06 (0.05, 0.07)	0.04 (0.03, 0.05)	17,719 (7,794, 30,921)	0.02 (0.02, 0.03)	0.02 (0.01, 0.02)	3,630 (-691, 8,807)	0.04 (0.03, 0.05)	0.03 (0.02, 0.04)	14,089 (5,291, 24,429)
Total	0.34 (0.31-0.38)	0.26 (0.21-0.29)	87,758 (55,912-13,085)	0.15 (0.13-0.18)	0.11 (0.09-0.13)	40,704 (24,412-61,350)	0.19 (0.17-0.22)	0.14 (0.11-0.17)	47,055 (26,811-75,635)

Data in the Table are the number of incident heart failure events prevented among non-Hispanic Black adults (95% confidence interval) with current systolic blood pressure levels, with systolic blood pressure equity and the difference between these numbers among men and women not taking antihypertensive medication (top panel) and taking antihypertensive medication (bottom panel).

SBP – systolic blood pressure

© 2025 Hardy ST et al. *JAMA Network Open*.

eTable 7. Number of Cardiovascular Disease Events Expected With SBP Equity Accounting for a 10% Decrease in Effectiveness of the Intervention Reported From the Blood Pressure–Lowering Treatment Trialists Collaboration

	Not taking antihypertensive medication								
CVD	Non-Hispanic Black with current SBP levels			Non-Hispanic Black with SBP equity			Difference in number of events: current SBP levels minus SBP equity		
Age, years	Women (Millions)	Men (Millions)	Total (Millions)	Women (Millions)	Men (Millions)	Total (Millions)	Women	Men	Total
45 – 64	0.09 (0.07-0.11)	0.16 (0.13-0.20)	0.25 (0.21-0.29)	0.08 (0.06-0.10)	0.15 (0.12-0.18)	0.22 (0.19-0.27)	9720 (5086 - 16399)	15410 (8099 - 24800)	25130 (15422 - 38727)
65 – 74	0.02 (0.02-0.03)	0.05 (0.04-0.06)	0.07 (0.06-0.09)	0.02 (0.01-0.03)	0.04 (0.03-0.05)	0.06 (0.05-0.07)	4803.3 (1945 - 8603)	7611.5 (3364 - 14269)	12415 (6874 - 20279)
>=75	0.02 (0.02-0.03)	0.03 (0.02-0.04)	0.05 (0.05-0.06)	0.02 (0.01-0.02)	0.03 (0.02-0.04)	0.05 (0.04-0.06)	3967.7 (-18 - 8420)	4224.2 (965 - 9214)	8192.0 (3086 - 14630)
Total	0.13 (0.11-0.16)	0.24 (0.21-0.28)	0.38 (0.33-0.42)	0.11 (0.09-0.14)	0.22 (0.18-0.25)	0.33 (0.29-0.38)	18491 (10811- 28800)	27246 (16666 - 41522)	45737 (30298 - 65930)
	**Taking antihypertensive medication**								
	**Non-Hispanic Black with current SBP levels**			**Non-Hispanic Black with SBP equity**			**Difference in number of events: current SBP levels minus SBP equity**		
**Age, years**	**Women (Millions)**	**Men (Millions)**	**Total (Millions)**	**Women (Millions)**	**Men (Millions)**	**Total (Millions)**	**Women**	**Men**	**Total**
45 – 64	0.25 (0.23-0.28)	0.24 (0.21-0.27)	0.49 (0.45-0.53)	0.22 (0.19-0.26)	0.20 (0.17-0.23)	0.42 (0.38-0.47)	27910 (12522-49095)	37217 (23586-55666)	65127 (40223.9-94099)
65 – 74	0.14 (0.13-0.16)	0.10 (0.08-0.11)	0.24 (0.22-0.26)	0.13 (0.11-0.15)	0.09 (0.07-0.10)	0.22 (0.19-0.24)	13521 (3495-27779)	9238 (814.9-19545)	22759.6 (9389.5-40447)
>=75	0.11 (0.09-0.12)	0.05 (0.04-0.06)	0.16 (0.14-0.18)	0.09 (0.07-0.11)	0.05 (0.04-0.06)	0.13 (0.11-0.15)	19132.5 (6896-34407)	4440 (-1022.1-11015)	23572 (10145-41014)
Total	0.50 (0.47-0.54)	0.39 (0.35-0.42)	0.89 (0.84-0.93)	0.44 (0.40-0.48)	0.34 (0.30-0.37)	0.78 (0.71-0.83)	60564.0 (35497-97082)	50894.6 (31196.2-76147)	111459 (73288-164600)

Data include current systolic blood pressure levels, with systolic blood pressure equity and the difference between these numbers.

© 2025 Hardy ST et al. *JAMA Network Open*.

eTable 8. Number of Coronary Heart Disease Events Expected With SBP Equity Accounting for a 10% Decrease in Effectiveness of the Intervention Reported From the Blood Pressure–Lowering Treatment Trialists Collaboration

	Not taking antihypertensive medication								
**CHD**	**Non-Hispanic Black with current SBP levels**			**Non-Hispanic Black with SBP equity**			**Difference in number of events: current SBP levels minus SBP equity**		
**Age, years**	**Women (Millions)**	**Men (Millions)**	**Total (Millions)**	**Women (Millions)**	**Men (Millions)**	**Total (Millions)**	**Women**	**Men**	**Total**
45 – 64	0.05 (0.04-0.07)	0.10 (0.07-0.13)	0.15 (0.11-0.18)	0.05 (0.03-0.06)	0.09 (0.06-0.13)	0.14 (0.11-0.17)	3134 (607-7448)	5053 (930-11523)	8187 (1465-17212)
65 – 74	0.01 (0.01-0.02)	0.03 (0.02-0.04)	0.04 (0.03-0.05)	0.01 (0.01-0.01)	0.03 (0.02-0.04)	0.04 (0.03-0.05)	1202 (240-3357)	2743 (615-7384)	3946 (785-9182)
>=75	0.01 (0.01-0.02)	0.02 (0.02-0.03)	0.03 (0.02-0.04)	0.01 (0.01-0.01)	0.02 (0.01-0.03)	0.03 (0.02-0.04)	1041 (42 - 3575)	1544 (176-4617)	2586 (358-6551)
Total	0.07 (0.05-0.09)	0.15 (0.12-0.19)	0.22 (0.18-0.26)	0.06 (0.05-0.08)	0.14 (0.11-0.17)	0.20 (0.17-0.25)	5377 (1108-11843)	9341 (1806-19658)	14719 (2310-29227)
	**Taking antihypertensive medication**								
	**Non-Hispanic Black with current SBP levels**			**Non-Hispanic Black with SBP equity**			**Difference in number of events: current SBP levels minus SBP equity**		
**Age, years**	**Women (Millions)**	**Men (Millions)**	**Total (Millions)**	**Women (Millions)**	**Men (Millions)**	**Total (Millions)**	**Women**	**Men**	**Total**
45 – 64	0.13 (0.11-0.15)	0.14 (0.12-0.17)	0.27 (0.24-0.30)	0.12 (0.10-0.14)	0.13 (0.11-0.15)	0.25 (0.22-0.28)	7978 (1845-20952)	12496 (2731-25526)	20474 (4274-41663)
65 – 74	0.07 (0.06-0.08)	0.06 (0.05-0.07)	0.14 (0.12-0.15)	0.07 (0.06-0.08)	0.06 (0.05-0.07)	0.13 (0.11-0.15)	3804 (673-11139)	3393 (354-10242)	7196 (1731-18642)
>=75	0.05 (0.04-0.06)	0.03 (0.02-0.03)	0.08 (0.07-0.09)	0.05 (0.04-0.06)	0.03 (0.02-0.03)	0.07 (0.06-0.09)	5376 (1067-13276)	1256 (-130 - 4426)	6632 (1385-16610)
Total	0.25 (0.23-0.28)	0.23 (0.21-0.26)	0.49 (0.45-0.52)	0.24 (0.21-0.27)	0.22 (0.19-0.24)	0.45 (0.41-0.49)	17158 (4215 - 40546)	17144 (3797-34542)	34302 (7708-69398)

Data include current systolic blood pressure levels, with systolic blood pressure equity and the difference between these numbers.

© 2025 Hardy ST et al. *JAMA Network Open*.

eTable 9. Number of Stroke Events Expected With SBP Equity Accounting for a 10% Decrease in Effectiveness of the Intervention Reported From the Blood Pressure–Lowering Treatment Trialists Collaboration

	**Not taking antihypertensive medication**								
**Stroke**	**Non-Hispanic Black with current SBP levels**			**Non-Hispanic Black with SBP equity**			**Difference in number of events: current SBP levels minus SBP equity**		
**Age, years**	**Women (Millions)**	**Men (Millions)**	**Total (Millions)**	**Women (Millions)**	**Men (Millions)**	**Total (Millions)**	**Women**	**Men**	**Total**
45 – 64	0.03 (0.02-0.04)	0.06 (0.04-0.08)	0.09 (0.06-0.11)	0.02 (0.01-0.04)	0.05 (0.03-0.07)	0.07 (0.05-0.09)	5305 (2586-10186)	8989 (4314-15756)	14294 (7992-23477)
65 – 74	0.01 (0.01-0.02)	0.01 (0.01-0.02)	0.02 (0.02-0.03)	0.01 (0.00-0.01)	0.01 (0.01-0.02)	0.02 (0.01-0.03)	3275 (1341-6933)	3439 (1552-7439)	6713.3 (3521-11844)
>=75	0.01 (0.01-0.01)	0.01 (0.01-0.01)	0.02 (0.01-0.02)	0.01 (0.00-0.01)	0.01 (0.00-0.01)	0.01 (0.01-0.02)	2453.1 (121-5519)	1954 (454-4992)	4407.4 (1452-8499)
Total	0.05 (0.04-0.06)	0.08 (0.06-0.10)	0.13 (0.10-0.15)	0.04 (0.03-0.05)	0.07 (0.05-0.09)	0.10 (0.08-0.13)	11032 (5985-18449)	14382 (7900-23522)	25415 (15065-38416)
	**Taking antihypertensive medication**								
	**Non-Hispanic Black with current SBP levels**			**Non-Hispanic Black with SBP equity**			**Difference in number of events: current SBP levels minus SBP equity**		
**Age, years**	**Women (Millions)**	**Men (Millions)**	**Total (Millions)**	**Women (Millions)**	**Men (Millions)**	**Total (Millions)**	**Women**	**Men**	**Total**
45 – 64	0.08 (0.06-0.10)	0.06 (0.05-0.08)	0.14 (0.12-0.17)	0.06 (0.05-0.08)	0.05 (0.04-0.06)	0.11 (0.09-0.13)	14347 (6258-26618)	16160 (9417-26005)	30506 (17366-47134)
65 – 74	0.05 (0.04-0.06)	0.03 (0.02-0.03)	0.08 (0.07-0.09)	0.04 (0.03-0.06)	0.02 (0.02-0.03)	0.07 (0.05-0.08)	8227 (2317-17775)	4039 (382-8931)	12266 (4919-22432)
>=75	0.04 (0.03-0.05)	0.02 (0.01-0.02)	0.05 (0.04-0.07)	0.03 (0.02-0.04)	0.01 (0.01-0.02)	0.04 (0.03-0.05)	11304 (4055-20523)	2169 (-384-5563)	13474 (5626-24363)
Total	0.17 (0.15-0.19)	0.10 (0.09-0.12)	0.27 (0.25-0.30)	0.14 (0.11-0.16)	0.08 (0.07-0.10)	0.22 (0.18-0.25)	33878 (18596-56424)	22368 (12899-35414)	56246 (34501-87024)

Data include current systolic blood pressure levels, with systolic blood pressure equity and the difference between these numbers.

© 2025 Hardy ST et al. *JAMA Network Open*.

eTable 10. Number of Heart Failure Events Expected With SBP Equity Accounting for a 10% Decrease in Effectiveness of the Intervention Reported From the Blood Pressure–Lowering Treatment Trialists Collaboration

	Not taking antihypertensive medication								
**Heart failure**	**Non-Hispanic Black with current SBP levels**			**Non-Hispanic Black with SBP equity**			**Difference in number of events: current SBP levels minus SBP equity**		
**Age, years**	**Women (Millions)**	**Men (Millions)**	**Total (Millions)**	**Women (Millions)**	**Men (Millions)**	**Total (Millions)**	**Women**	**Men**	**Total**
45 – 64	0.03 (0.02-0.05)	0.03 (0.02-0.06)	0.06 (0.04-0.09)	0.02 (0.01-0.04)	0.03 (0.01-0.05)	0.05 (0.03-0.07)	5744 (2653-11993)	5411 (2303.2-11455.6)	11155 (5821.9-19806.3)
65 – 74	0.01 (0.00-0.01)	0.01 (0.01-0.02)	0.02 (0.02-0.03)	0.00 (0.00-0.01)	0.01 (0.01-0.02)	0.01 (0.01-0.02)	1998 (807-4537)	4225 (1935.3-8500.6)	6222 (3181-11284)
>=75	0.01 (0.00-0.01)	0.01 (0.01-0.02)	0.02 (0.01-0.02)	0.00 (0.00-0.01)	0.01 (0.00-0.01)	0.01 (0.01-0.02)	1807 (130-4398)	2535 (611.5-5921.3)	4342 (1578.9-8455.9)
Total	0.04 (0.03-0.06)	0.06 (0.04-0.08)	0.09 (0.07-0.12)	0.03 (0.02-0.04)	0.04 (0.03-0.06)	0.07 (0.05-0.10)	9549 (4960-17269)	12170 (6381.1-20676.9)	21719 (11775-33803)
	**Taking antihypertensive medication**								
	**Non-Hispanic Black with current SBP levels**			**Non-Hispanic Black with SBP equity**			**Difference in number of events: current SBP levels minus SBP equity**		
**Age, years**	**Women (Millions)**	**Men (Millions)**	**Total (Millions)**	**Women (Millions)**	**Men (Millions)**	**Total (Millions)**	**Women**	**Men**	**Total**
45 – 64	0.10 (0.08-0.12)	0.10 (0.08-0.12)	0.19 (0.17-0.22)	0.08 (0.06-0.10)	0.07 (0.05-0.09)	0.15 (0.12-0.18)	20198 (8805-37370)	27616 (15997-42521)	47814 (27398-72495)
65 – 74	0.06 (0.05-0.06)	0.03 (0.03-0.04)	0.09 (0.08-0.10)	0.05 (0.03-0.06)	0.03 (0.02-0.04)	0.07 (0.06-0.09)	9823 (2743-20889)	6295.7 (609-13730)	16119 (6691-29373)
>=75	0.04 (0.03-0.05)	0.02 (0.02-0.03)	0.06 (0.05-0.07)	0.03 (0.02-0.04)	0.02 (0.01-0.02)	0.04 (0.03-0.06)	12942 (4686-23268)	3298 (-594-8275)	16239 (6845-29396)
Total	0.19 (0.17-0.22)	0.15 (0.13-0.17)	0.34 (0.31-0.37)	0.15 (0.12-0.18)	0.12 (0.09-0.14)	0.26 (0.22-0.30)	42963 (23558-71846)	37210 (21595-57378)	80173 (48384-121614)

Data include current systolic blood pressure levels, with systolic blood pressure equity and the difference between these numbers.

© 2025 Hardy ST et al. *JAMA Network Open*.

eTable 11. Number of Cardiovascular Mortality Events Expected With SBP Equity Accounting for a 10% Decrease in Effectiveness of the Intervention Reported From the Blood Pressure–Lowering Treatment Trialists Collaboration

	**Not taking antihypertensive medication**								
**CVD mortality**	**Non-Hispanic Black with current SBP levels**			**Non-Hispanic Black with SBP equity**			**Difference in number of events: current SBP levels minus SBP equity**		
**Age, years**	**Women (Millions)**	**Men (Millions)**	**Total (Millions)**	**Women (Millions)**	**Men (Millions)**	**Total (Millions)**	**Women**	**Men**	**Total**
45 – 64	0.03 (0.02-0.04)	0.06 (0.04-0.09)	0.09 (0.07-0.12)	0.03 (0.02-0.04)	0.06 (0.04-0.09)	0.08 (0.06-0.11)	2517 (653-6035)	4680 (1212-10352)	7196 (1683-14969)
65 – 74	0.02 (0.01-0.02)	0.03 (0.02-0.04)	0.05 (0.04-0.06)	0.01 (0.01-0.02)	0.03 (0.02-0.04)	0.04 (0.03-0.05)	2461 (608-6208)	3577 (957-8657)	6039 (1497-12713)
>=75	0.02 (0.02-0.03)	0.03 (0.02-0.04)	0.05 (0.04-0.06)	0.02 (0.01-0.03)	0.03 (0.02-0.04)	0.05 (0.04-0.06)	3107 (120-8994)	3316 (492-9064)	6422 (1212-14801)
Total	0.07 (0.05-0.08)	0.13 (0.10-0.16)	0.19 (0.16-0.22)	0.06 (0.04-0.07)	0.11 (0.09-0.14)	0.17 (0.14-0.21)	8085 (2148-17158)	11573 (3011-23263)	19657 (5019-38110)
	**Taking antihypertensive medication**								
	**Non-Hispanic Black with current SBP levels**			**Non-Hispanic Black with SBP equity**			**Difference in number of events: current SBP levels minus SBP equity**		
**Age, years**	**Women (Millions)**	**Men (Millions)**	**Total (Millions)**	**Women (Millions)**	**Men (Millions)**	**Total (Millions)**	**Women**	**Men**	**Total**
45 – 64	0.10 (0.09-0.12)	0.13 (0.11-0.16)	0.24 (0.21-0.27)	0.09 (0.08-0.12)	0.12 (0.09-0.15)	0.21 (0.18-0.25)	9026 (2554-22150)	16427 (4409-31685)	25452 (7037.6-48730)
65 – 74	0.07 (0.06-0.08)	0.06 (0.05-0.07)	0.13 (0.11-0.14)	0.06 (0.05-0.08)	0.05 (0.04-0.07)	0.12 (0.10-0.13)	4909 (1093-13926)	4391 (572-12446)	9300.6 (2588-22502)
>=75	0.08 (0.07-0.10)	0.05 (0.04-0.06)	0.13 (0.11-0.15)	0.07 (0.06-0.09)	0.04 (0.04-0.05)	0.12 (0.09-0.14)	12035 (2668-27166)	3072 (-309-10006)	15107 (4137-35469)
Total	0.26 (0.23-0.28)	0.24 (0.21-0.27)	0.50 (0.46-0.53)	0.23 (0.20-0.26)	0.22 (0.18-0.25)	0.45 (0.39-0.50)	25970 (7667-54414)	23890 (7577-46602)	49860 (14915-95063)

Data include current systolic blood pressure levels, with systolic blood pressure equity and the difference between these numbers.

© 2025 Hardy ST et al. *JAMA Network Open*.

eTable 12. Number of Cardiovascular Disease Events Expected With SBP Equity Accounting for a 10% Increase in Effectiveness of the Intervention Reported From the Blood Pressure–Lowering Treatment Trialists Collaboration

	Not taking antihypertensive medication								
**CVD**	**Non-Hispanic Black with current SBP levels**			**Non-Hispanic Black with SBP equity**			**Difference in number of events: current SBP levels minus SBP equity**		
**Age, years**	**Women (Millions)**	**Men (Millions)**	**Total (Millions)**	**Women (Millions)**	**Men (Millions)**	**Total (Millions)**	**Women**	**Men**	**Total**
45 – 64	0.09 (0.07-0.11)	0.16 (0.13-0.20)	0.25 (0.21-0.29)	0.07 (0.06-0.10)	0.14 (0.12-0.18)	0.22 (0.18-0.26)	11724 (6441-19224)	18632 (9915-28854)	30357 (20131-45527)
65 – 74	0.02 (0.02-0.03)	0.05 (0.04-0.06)	0.07 (0.06-0.09)	0.02 (0.01-0.03)	0.04 (0.03-0.05)	0.06 (0.05-0.07)	5737 (2438-9906)	9135 (4093-16408)	14872 (8541-23446)
>=75	0.02 (0.02-0.03)	0.03 (0.02-0.04)	0.05 (0.05-0.06)	0.02 (0.01-0.02)	0.03 (0.02-0.04)	0.04 (0.03-0.05)	4746 (-37-9668)	5084 (1187-10601)	9831 (3846-17103)
Total	0.13 (0.11-0.16)	0.24 (0.21-0.28)	0.38 (0.33-0.42)	0.11 (0.09-0.13)	0.21 (0.18-0.25)	0.32 (0.28-0.37)	22208 (13530-33400)	32852 (21153-48430)	55059 (37843-76039)
	**Taking antihypertensive medication**								
	**Non-Hispanic Black with current SBP levels**			**Non-Hispanic Black with SBP equity**			**Difference in number of events: current SBP levels minus SBP equity**		
**Age, years**	**Women (Millions)**	**Men (Millions)**	**Total (Millions)**	**Women (Millions)**	**Men (Millions)**	**Total (Millions)**	**Women**	**Men**	**Total**
45 – 64	0.25 (0.23-0.28)	0.24 (0.21-0.27)	0.49 (0.45-0.53)	0.22 (0.19-0.25)	0.19 (0.17-0.23)	0.41 (0.37-0.45)	33678 (15481-57110)	44666 (28619-64368)	78344 (51107-109255)
65 – 74	0.14 (0.13-0.16)	0.10 (0.08-0.11)	0.24 (0.22-0.26)	0.13 (0.11-0.15)	0.09 (0.07-0.10)	0.21 (0.19-0.24)	16348 (4244-32496)	11168 (731-22728)	27516 (11477-47121)
>=75	0.11 (0.09-0.12)	0.05 (0.04-0.06)	0.16 (0.14-0.18)	0.08 (0.06-0.10)	0.05 (0.04-0.06)	0.13 (0.11-0.15)	22889 (8588-39347)	5374 (-1353-12893)	28263 (12407-46987)
Total	0.50 (0.47-0.54)	0.39 (0.35-0.42)	0.89 (0.84-0.93)	0.43 (0.38-0.47)	0.33 (0.29-0.36)	0.75 (0.69-0.81)	72915 (44623-112403)	61208 (39638-89145)	134123 (93784-188198)

Data include current systolic blood pressure levels, with systolic blood pressure equity and the difference between these numbers.

© 2025 Hardy ST et al. *JAMA Network Open*.

eTable 13. Number of Coronary Heart Disease Events Expected With SBP Equity Accounting for a 10% Increase in Effectiveness of the Intervention Reported From the Blood Pressure–Lowering Treatment Trialists Collaboration

	Not taking antihypertensive medication								
**CHD**	**Non-Hispanic Black with current SBP levels**			**Non-Hispanic Black with SBP equity**			**Difference in number of events: current SBP levels minus SBP equity**		
**Age, years**	**Women (Millions)**	**Men (Millions)**	**Total (Millions)**	**Women (Millions)**	**Men (Millions)**	**Total (Millions)**	**Women**	**Men**	**Total**
45 – 64	0.05 (0.04-0.07)	0.10 (0.07-0.13)	0.15 (0.11-0.18)	0.05 (0.03-0.06)	0.09 (0.06-0.13)	0.14 (0.10-0.17)	3802 (1176-8346)	6140 (1916-12976)	9942 (3005-19440)
65 – 74	0.01 (0.01-0.02)	0.03 (0.02-0.04)	0.04 (0.03-0.05)	0.01 (0.01-0.01)	0.03 (0.02-0.04)	0.04 (0.03-0.05)	1451 (407-3632)	3320 (1025-7896)	4771 (1545-10164)
>=75	0.01 (0.01-0.02)	0.02 (0.02-0.03)	0.03 (0.02-0.04)	0.01 (0.01-0.01)	0.02 (0.01-0.03)	0.03 (0.02-0.04)	1258 (65-3804)	1872 (323-5188)	3129 (726-7328)
Total	0.07 (0.05-0.09)	0.15 (0.12-0.19)	0.22 (0.18-0.26)	0.06 (0.05-0.08)	0.14 (0.11-0.17)	0.20 (0.16-0.24)	6511 (2079-13107)	11331 (3778-21999)	17842 (5513-32701)
	**Taking antihypertensive medication**								
	**Non-Hispanic Black with current SBP levels**			**Non-Hispanic Black with SBP equity**			**Difference in number of events: current SBP levels minus SBP equity**		
**Age, years**	**Women (Millions)**	**Men (Millions)**	**Total (Millions)**	**Women (Millions)**	**Men (Millions)**	**Total (Millions)**	**Women**	**Men**	**Total**
45 – 64	0.13 (0.11-0.15)	0.14 (0.12-0.17)	0.27 (0.24-0.30)	0.12 (0.10-0.14)	0.13 (0.11-0.15)	0.25 (0.21-0.28)	9683 (3007-22432.1)	15120 (5099-27617)	24803 (8261-45895)
65 – 74	0.07 (0.06-0.08)	0.06 (0.05-0.07)	0.14 (0.12-0.15)	0.07 (0.06-0.08)	0.06 (0.05-0.07)	0.13 (0.11-0.14)	4621 (1099-12691)	4122 (502-11382)	8743 (2670-20514)
>=75	0.05 (0.04-0.06)	0.03 (0.02-0.03)	0.08 (0.07-0.09)	0.05 (0.04-0.06)	0.03 (0.02-0.03)	0.07 (0.06-0.08)	6494 (1667-14638)	1527 (-155-4954)	8020 (2438-18353)
Total	0.25 (0.23-0.28)	0.23 (0.21-0.26)	0.49 (0.45-0.52)	0.23 (0.20-0.26)	0.21 (0.18-0.24)	0.44 (0.40-0.49)	20797 (7290-44277)	20768 (6833-38849)	41566 (15010-77439)

Data include current systolic blood pressure levels, with systolic blood pressure equity and the difference between these numbers.

© 2025 Hardy ST et al. *JAMA Network Open*.

eTable 14. Number of Stroke Events Expected With SBP Equity Accounting for a 10% Increase in Effectiveness of the Intervention Reported From the Blood Pressure–Lowering Treatment Trialists Collaboration

	**Not taking antihypertensive medication**								
**Stroke**	**Non-Hispanic Black with current SBP levels**			**Non-Hispanic Black with SBP equity**			**Difference in number of events: current SBP levels minus SBP equity**		
**Age, years**	**Women (Millions)**	**Men (Millions)**	**Total (Millions)**	**Women (Millions)**	**Men (Millions)**	**Total (Millions)**	**Women**	**Men**	**Total**
45 – 64	0.03 (0.02-0.04)	0.06 (0.04-0.08)	0.09 (0.06-0.11)	0.02 (0.01-0.03)	0.05 (0.03-0.07)	0.07 (0.05-0.09)	6464 (3370-11949)	11002 (5669-18803)	17466 (10741-27752)
65 – 74	0.01 (0.01-0.02)	0.01 (0.01-0.02)	0.02 (0.02-0.03)	0.01 (0.00-0.01)	0.01 (0.01-0.02)	0.02 (0.01-0.02)	3918 (1797-8106)	415 (1917-8519)	8079 (4529-13717)
>=75	0.01 (0.01-0.01)	0.01 (0.01-0.01)	0.02 (0.01-0.02)	0.01 (0.00-0.01)	0.01 (0.00-0.01)	0.01 (0.01-0.02)	2944 (282-6372)	2372 (607-5687)	5315 (1867-9770)
Total	0.05 (0.04-0.06)	0.08 (0.06-0.10)	0.13 (0.10-0.15)	0.03 (0.02-0.05)	0.06 (0.04-0.08)	0.10 (0.07-0.12)	13326 (7794-21506)	17525 (10575-28322)	30851 (20636-45541)
	**Taking antihypertensive medication**								
	**Non-Hispanic Black with current SBP levels**			**Non-Hispanic Black with SBP equity**			**Difference in number of events: current SBP levels minus SBP equity**		
**Age, years**	**Women (Millions)**	**Men (Millions)**	**Total (Millions)**	**Women (Millions)**	**Men (Millions)**	**Total (Millions)**	**Women**	**Men**	**Total**
45 – 64	0.08 (0.06-0.10)	0.06 (0.05-0.08)	0.14 (0.12-0.17)	0.06 (0.04-0.08)	0.04 (0.03-0.06)	0.10 (0.08-0.13)	17493 (8239-31357)	19508 (12371-30041)	37001 (23179-55526)
65 – 74	0.05 (0.04-0.06)	0.03 (0.02-0.03)	0.08 (0.07-0.09)	0.04 (0.03-0.05)	0.02 (0.01-0.03)	0.06 (0.05-0.08)	10069 (2956-20838)	4942 (554-10428)	15011 (6504-26004)
>=75	0.04 (0.03-0.05)	0.02 (0.01-0.02)	0.05 (0.04-0.07)	0.03 (0.02-0.04)	0.01 (0.01-0.02)	0.04 (0.03-0.05)	13568 (5633-23480)	2661 (-470-6486.)	16228 (7505-27966)
Total	0.17 (0.15-0.19)	0.10 (0.09-0.12)	0.27 (0.25-0.30)	0.13 (0.10-0.15)	0.08 (0.06-0.09)	0.21 (0.17-0.23)	41130 (25088-64910)	27111 (17336-41378)	68241 (46790-101078)

Data include current systolic blood pressure levels, with systolic blood pressure equity and the difference between these numbers.

© 2025 Hardy ST et al. *JAMA Network Open*.

eTable 15. Number of Heart Failure Events Expected With SBP Equity Accounting for a 10% Increase in Effectiveness of the Intervention Reported From the Blood Pressure–Lowering Treatment Trialists Collaboration

	**Not taking antihypertensive medication**								
**Heart failure**	**Non-Hispanic Black with current SBP levels**			**Non-Hispanic Black with SBP equity**			**Difference in number of events: current SBP levels minus SBP equity**		
**Age, years**	**Women (Millions)**	**Men (Millions)**	**Total (Millions)**	**Women (Millions)**	**Men (Millions)**	**Total (Millions)**	**Women**	**Men**	**Total**
45 – 64	0.03 (0.02-0.05)	0.03 (0.02-0.06)	0.06 (0.04-0.09)	0.02 (0.01-0.04)	0.02 (0.01-0.05)	0.04 (0.03-0.07)	6844 (3345-13744)	6476 (2871-13022)	13320 (7371-23149)
65 – 74	0.01 (0.00-0.01)	0.01 (0.01-0.02)	0.02 (0.02-0.03)	0.00 (0.00-0.01)	0.01 (0.01-0.02)	0.01 (0.01-0.02)	2338 (1006-5178)	4986 (2418-9605)	7324 (3927-12616)
>=75	0.01 (0.00-0.01)	0.01 (0.01-0.02)	0.02 (0.01-0.02)	0.00 (0.00-0.01)	0.01 (0.00-0.01)	0.01 (0.01-0.02)	2119 (228-5078)	3008 (771-6596)	5128 (1966-9448)
Total	0.04 (0.03-0.06)	0.06 (0.04-0.08)	0.09 (0.07-0.12)	0.03 (0.02-0.04)	0.04 (0.03-0.06)	0.07 (0.05-0.09)	11302 (6293-19771)	14470 (8061-23866)	25772 (15805-38313)
	**Taking antihypertensive medication**								
	**Non-Hispanic Black with current SBP levels**			**Non-Hispanic Black with SBP equity**			**Difference in number of events: current SBP levels minus SBP equity**		
**Age, years**	**Women (Millions)**	**Men (Millions)**	**Total (Millions)**	**Women (Millions)**	**Men (Millions)**	**Total (Millions)**	**Women**	**Men**	**Total**
45 – 64	0.10 (0.08-0.12)	0.10 (0.08-0.12)	0.19 (0.17-0.22)	0.07 (0.05-0.10)	0.06 (0.05-0.08)	0.14 (0.11-0.17)	24082 (10858-41919)	32597 (20662-48440)	56679 (34699-81891)
65 – 74	0.06 (0.05-0.06)	0.03 (0.03-0.04)	0.09 (0.08-0.10)	0.04 (0.03-0.06)	0.03 (0.02-0.04)	0.07 (0.06-0.09)	11757 (3370-23917)	7533 (739-15598)	19290 (8329-32995)
>=75	0.04 (0.03-0.05)	0.02 (0.02-0.03)	0.06 (0.05-0.07)	0.02 (0.01-0.04)	0.02 (0.01-0.02)	0.04 (0.03-0.05)	15187 (5863-26168)	3955 (-767-9423)	19142 (8779-32784)
Total	0.19 (0.17-0.22)	0.15 (0.13-0.17)	0.34 (0.31-0.37)	0.14 (0.11-0.17)	0.11 (0.09-0.13)	0.25 (0.21-0.29)	51026 (29952-79983)	44085 (27278-64746)	95111 (62579-137373)

Data include current systolic blood pressure levels, with systolic blood pressure equity and the difference between these numbers.

eTable 16. Number of Cardiovascular Mortality Events Expected With SBP Equity Accounting for a 10% Increase in Effectiveness of the Intervention Reported From the Blood Pressure–Lowering Treatment Trialists Collaboration

	Not taking antihypertensive medication								
**CVD mortality**	**Non-Hispanic Black with current SBP levels**			**Non-Hispanic Black with SBP equity**			**Difference in number of events: current SBP levels minus SBP equity**		
**Age, years**	**Women (Millions)**	**Men (Millions)**	**Total (Millions)**	**Women (Millions)**	**Men (Millions)**	**Total (Millions)**	**Women**	**Men**	**Total**
45 – 64	0.03 (0.02-0.04)	0.06 (0.04-0.09)	0.09 (0.07-0.12)	0.03 (0.02-0.04)	0.06 (0.04-0.09)	0.08 (0.06-0.11)	3045 (1073-6695)	5672 (2018-11643)	8717 (3457-17038)
65 – 74	0.02 (0.01-0.02)	0.03 (0.02-0.04)	0.05 (0.04-0.06)	0.01 (0.01-0.02)	0.02 (0.02-0.03)	0.04 (0.03-0.05)	2955 (924-6724)	4311 (1473-9919)	7266 (2854-14691)
>=75	0.02 (0.02-0.03)	0.03 (0.02-0.04)	0.05 (0.04-0.06)	0.02 (0.01-0.03)	0.03 (0.02-0.04)	0.05 (0.04-0.06)	3734 (214-10004)	4004 (769-10201)	7739 (2129-16511)
Total	0.07 (0.05-0.08)	0.13 (0.10-0.16)	0.19 (0.16-0.22)	0.06 (0.04-0.07)	0.11 (0.09-0.14)	0.17 (0.13-0.20)	9734 (3626-19031)	13988 (5299-26292)	23721 (9347-42320)
	**Taking antihypertensive medication**								
	**Non-Hispanic Black with current SBP levels**			**Non-Hispanic Black with SBP equity**			**Difference in number of events: current SBP levels minus SBP equity**		
**Age, years**	**Women (Millions)**	**Men (Millions)**	**Total (Millions)**	**Women (Millions)**	**Men (Millions)**	**Total (Millions)**	**Women**	**Men**	**Total**
45 – 64	0.10 (0.09-0.12)	0.13 (0.11-0.16)	0.24 (0.21-0.27)	0.09 (0.08-0.11)	0.11 (0.09-0.14)	0.21 (0.17-0.24)	10922 (3701-24279)	19795 (7905-35774)	30718 (12012-54328)
65 – 74	0.07 (0.06-0.08)	0.06 (0.05-0.07)	0.13 (0.11-0.14)	0.06 (0.05-0.07)	0.05 (0.04-0.06)	0.11 (0.10-0.13)	5950 (1439-15438)	5322 (674-13842)	11272 (3794-24809)
>=75	0.08 (0.07-0.10)	0.05 (0.04-0.06)	0.13 (0.11-0.15)	0.07 (0.05-0.09)	0.04 (0.03-0.05)	0.11 (0.09-0.13)	14467 (4000-30383)	3727 (-420-11002)	18194 (6117-39109)
Total	0.26 (0.23-0.28)	0.24 (0.21-0.27)	0.50 (0.46-0.53)	0.22 (0.19-0.26)	0.21 (0.18-0.24)	0.43 (0.38-0.48)	31340 (12417-60581)	28844 (12192-52036)	60184 (2468-105826)

Data include current systolic blood pressure levels, with systolic blood pressure equity and the difference between these numbers.

© 2025 Hardy ST et al. *JAMA Network Open*.

eFigure 1. NHANES Study Inclusion Criteria

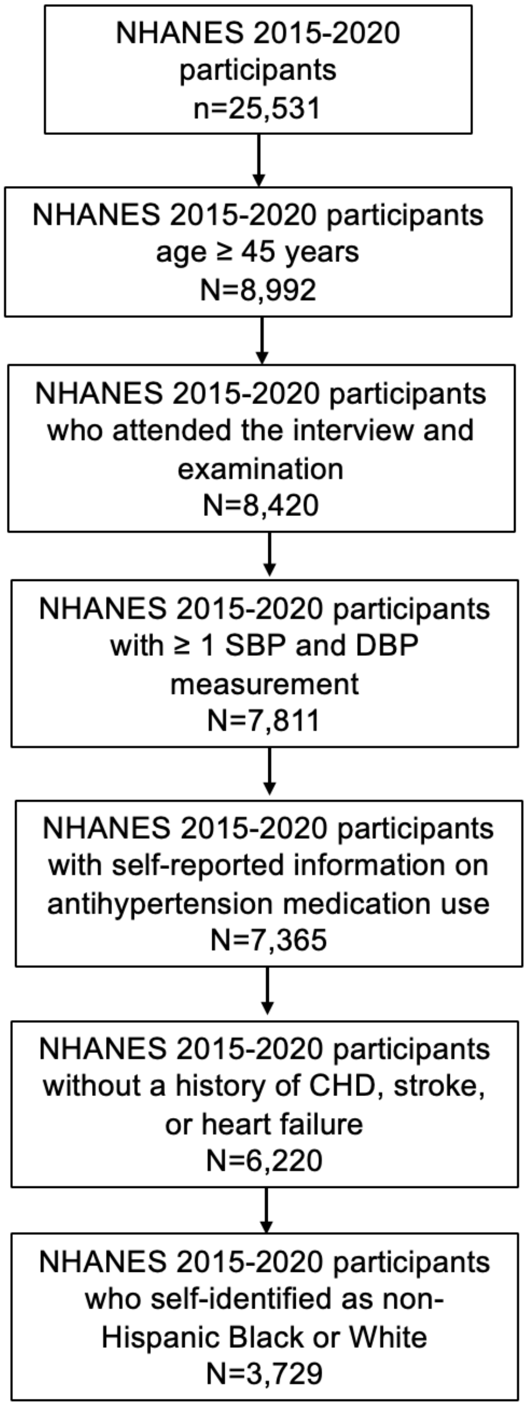

CHD – coronary heart disease; DBP – diastolic blood pressure; NHANES – National Health and Nutrition Examination Survey; SBP – systolic blood pressure

© 2025 Hardy ST et al. *JAMA Network Open*.

eFigure 2. REGARDS Study Inclusion Criteria

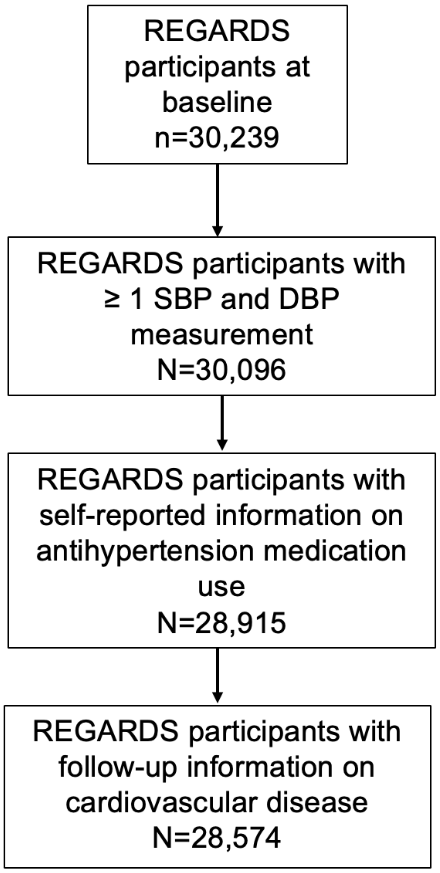

DBP – Diastolic blood pressure; REGARDS - Reasons for Geographic and Racial Differences in Stroke study; SBP – systolic blood pressure

© 2025 Hardy ST et al. *JAMA Network Open*.

eFigure 3. Number of Cardiovascular Disease Deaths That Could Be Prevented Among Non-Hispanic Black Adults With Systolic Blood Pressure Equity Between Non-Hispanic Black and White Adults Not Taking and Taking Antihypertensive Medication by Sex and Age

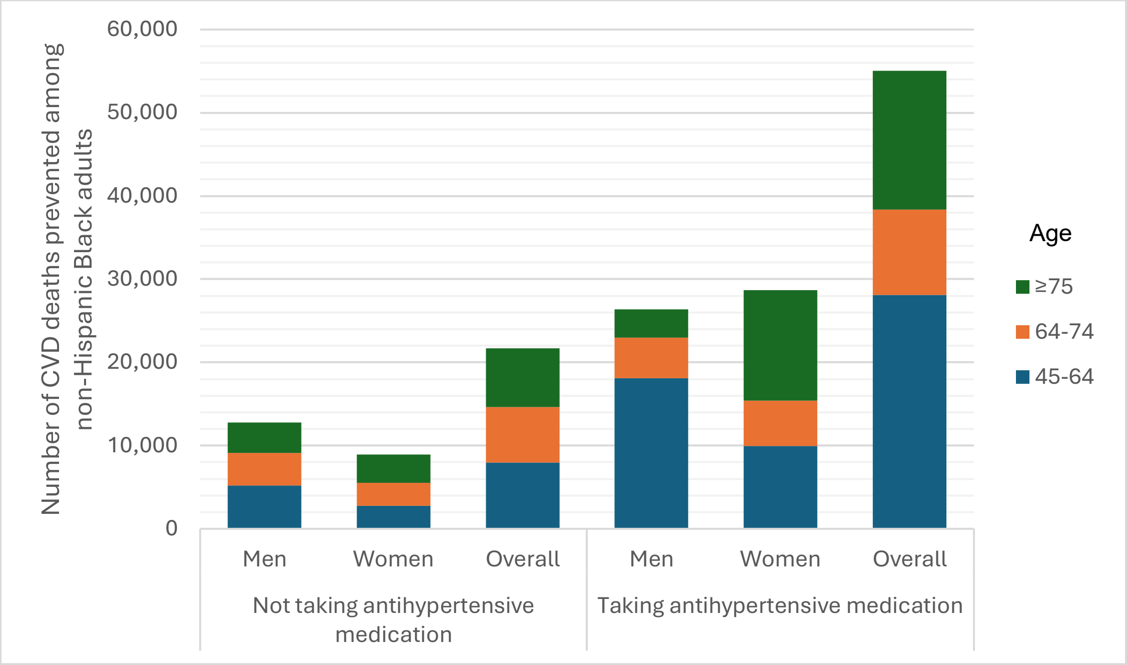

CVD, Cardiovascular disease

© 2025 Hardy ST et al. *JAMA Network Open*.
