# Supplementary material for: Prevention of Cardiovascular Disease Events and Deaths Among Black Adults Via Systolic Blood Pressure Equity
Source: JAMA Netw Open. 2025 Nov 4;8(11):e2541336. doi: 10.1001/jamanetworkopen.2025.41336 (PMC12587195; doi:10.1001/jamanetworkopen.2025.41336)
Supplement: Supplement 2. — Data Sharing Statement [file jamanetwopen-e2541336-s002.pdf]

Data Sharing Statement

Hardy. Prevention of Cardiovascular Disease Events and Deaths Among Black Adults Via Systolic Blood Pressure Equity. *JAMA Netw Open*. Published November 04, 2025. doi:10.1001/jamanetworkopen.2025.41336

Data
Data available: No

Additional Information
Explanation for why data not available: REGARDS data is available with an approved manuscript proposal. NHANES data is publically available via the NHANES website.
